# Numerical solution of DGLAP equations using Laguerre polynomials expansion and Monte Carlo method

**DOI:** 10.1186/s40064-016-3254-6

**Published:** 2016-09-29

**Authors:** A. Ghasempour Nesheli, A. Mirjalili, M. M. Yazdanpanah

**Affiliations:** 1 Department of Physics, Shiraz Branch, Islamic Azad University, Shiraz, Iran; 2 Physics Department, Yazd University, 89195-741 Yazd, Iran; 3 Faculty of Physics, Shahid Bahonar University of Kerman, Kerman, Iran

**Keywords:** DGLAP evolution equations, Evolved parton densities, Laguerre expansion, Monte Carlo method

## Abstract

We investigate the numerical solutions of the DGLAP evolution equations at the LO and NLO approximations, using the Laguerre polynomials expansion. The theoretical framework is based on Furmanski et al.’s articles. What makes the content of this paper different from other works, is that all calculations in the whole stages to extract the evolved parton distributions, are done numerically. The employed techniques to do the numerical solutions, based on Monte Carlo method, has this feature that all the results are obtained in a proper wall clock time by computer. The algorithms are implemented in FORTRAN and the employed coding ideas can be used in other numerical computations as well. Our results for the evolved parton densities are in good agreement with some phenomenological models. They also indicate better behavior with respect to the results of similar numerical calculations.

## Background

In the theory of strong interaction, the lepton–nucleon deep-inelastic scatterings (DIS) could lead us to get the required information for nucleon structure function. The DIS processes form the backbone of our knowledge for the parton densities, which are indispensable for analyses of hard scattering processes at proton–(anti-)proton colliders. Moreover, many experimental groups (Bloom et al. 1969; Breidenbach et al. 1969; Abbott et al. 1979) have observed the scaling behavior of the proton structure function in DIS (Bjorken 1969). This observation established the quark-parton model as a valid framework for interpreting DIS data; the DIS processes can be expressed in terms of universal parton densities. In Quantum Chromodynamics (QCD), structure functions are defined as convolutions of the universal parton momentum distributions inside the proton with the coefficient functions, which contain information about the boson–parton interaction. At large momentum transfers, $Q^{2} \gg 0$, the perturbative calculations of the coefficient functions predict a logarithmic dependence of the proton structure functions on *Q*^2^ to higher orders in *α*_*s*_. Thus, measurements of the structure functions allow precision tests of perturbative QCD. The standard and the basic tools for theoretical investigation of DIS structure functions are the DGLAP evolution equations (Gribov and Lipatov 1972; Dokshitzer 1977; Altarelli and Parisi 1977).

There exist several analytical and numerical methods to solve DGLAP evolution equations. What we present in this article, is a solution which is based entirely on numerical analyses of these equations, forming a series of Laguerre polynomials with respect to the $y = \ln (1/x)$ variable, where x is the fraction of proton momentum carried by the parton, The Laguerre series converges very quickly and it can easily be truncated with a reasonable precision.

In this article, we assume that reader is familiar with the required relations and the theoretical frameworks based on which the DGLAP evolution equations are working. So, at different sections of this article, we are mostly focused on presenting numerical investigations which would finally yield the evolved parton densities at energy scale *Q*^2^. In each section of the paper, we not only introduce the required theoretical expressions but also explain how to use them in practice to do our numerical calculations. The Monte Carlo algorithms which we construct are such as to make the wall clock time by computer in a proper time. The numerical patterns which we develop in this paper can also be used for other numerical investigations.

The organization of this paper is as following. In section “A short overview of the theoretical framework” we give a short overview on the evolution of parton densities, using DGLAP equations. The theoretical framework is based on using Laguerre polynomial expansions. In section “Basic tools, Monte Carlo solutions” the general structure of Monte Carlo algorithm is introduced. The required functions and subroutines are also introduced there. They can be requested via E-mail, a.mirjalili@yazd.ac.ir, from the authors. Then we use them to build the related Monte Carlo algorithm to get the numerical solutions for DGLAP evolution equations. The results are presented for the evolved parton densities at the end of section “Basic tools, Monte Carlo solutions” and also in sections “The programs and the results” and “Conclusion”, based on the numerically Monte Carlo algorithm. The results are in good agreement with the results of CTEQ and GRV parameterization groups. Finally, we give our conclusion in section 6.

## A short overview of the theoretical framework

In high energy physics, the parton densities at the *Q*^2^ scale can be obtained, employing the DGLAP evolution equations. These equations can be used to describe Bjorken violation in deep inelastic scattering (DIS). There are many different ways to solve DGLAP equations numerically. One of them is to use the Laguerre polynomial expansion which we employ to get the solution of non-singlet and singlet sectors of parton densities. To numerically achieve the evolved parton densities, we initially need to reach high levels of precision. The Laguerre polynomial expansion are rapidly convergent for medium values of *x* and at all energy scales. At very low*x*, say *x* < 0.001, these polynomials are numerically instable due to rapid rising of the splitting moments.

Following, we have provided the required definitions and conventions for the above mentioned numerical calculations:

### Running coupling constant

The *Q*^2^ dependence of the strong coupling constant *α*(*Q*^2^), considering the renormalization group equation, is given by:

1 $$Q^{2} \frac{d}{{dQ^{2} }}\alpha (Q^{2} ) = - \alpha (Q^{2} )\bar{\beta }\left[ {\alpha (Q^{2} )} \right]$$

where

2 $$\bar{\beta }(\alpha ) = \beta_{0} \frac{\alpha }{4\pi } + \beta_{1} \left( {\frac{\alpha }{4\pi }} \right)^{2} + \cdots ,$$

in which *β*_0_ and *β*_1_ are universal scheme independent coefficients and are given by:

3 $$\begin{aligned} \beta_{0} & = \frac{11}{3}C_{G} - \frac{4}{3}T_{R} n_{f} , \\ \beta_{1} & = \frac{34}{3}C_{G}^{2} - \frac{10}{3}C_{G} n_{f} - 2C_{F} n_{f} , \\ \end{aligned}$$

and $C_{G} = N,\;{\text{C}}_{\text{F}} = \frac{{{\text{N}}^{ 2} - 1}}{{ 2 {\text{N}}}},\;T_{R} = \frac{1}{2}$ where *N*and *n*_*f*_ refers respectively to the number of quark colors and flavors. The solution of *β*- QCD evolution equation, Eq. (1), at the next-to-leading order (NLO) approximation is given by:

4 $$\frac{{\alpha (Q^{2} )}}{2\pi } = \frac{2}{{\beta_{0} }}{\mkern 1mu} \frac{1}{{\ln {{Q^{2} } \mathord{\left/ {\vphantom {{Q^{2} } {\Lambda^{2} }}} \right. \kern-0pt} {\Lambda^{2} }}}}\left[ {1 - \frac{{\beta_{1} }}{{\beta_{0}^{2} }}\frac{{\ln \ln {{Q^{2} } \mathord{\left/ {\vphantom {{Q^{2} } {\Lambda^{2} }}} \right. \kern-0pt} {\Lambda^{2} }}}}{{\ln {{Q^{2} } \mathord{\left/ {\vphantom {{Q^{2} } {\Lambda^{2} }}} \right. \kern-0pt} {\Lambda^{2} }}}} + O\left( {\frac{1}{{\ln^{2} {{Q^{2} } \mathord{\left/ {\vphantom {{Q^{2} } {\Lambda^{2} }}} \right. \kern-0pt} {\Lambda^{2} }}}}} \right)} \right].$$

The cutoff parameter Λ, is determined by fitting the experimental data which at the NLO approximation is lower than 250 MeV.

### DGLAP evolution equations

Considering the contribution of quark-antiquark pair in evolution of quark densities, one would find out that for each quark flavor *i* with *i* = 1…2*n*_*f*_ summing over all quark and antiquark flavors, using the notations of Furmanski and Petroznio (1982a, b) we would have

5 $$q_{i}^{( + )} \equiv q_{i} + \bar{q}_{i} ,\quad q_{i}^{( - )} \equiv q_{i}^{V} \equiv q_{i} - \bar{q}_{i} ,\quad q^{( + )} \equiv \Sigma {\mkern 1mu} \equiv \sum\limits_{i = 1}^{{n_{f} }} {q_{i}^{( + )} } ,$$

Following that by defining

6 $$\chi_{i} (x,Q^{2} ) = q_{i}^{( + )} (x,Q^{2} ) - \frac{1}{{n_{f} }}q^{( + )} (x,Q^{2} ),$$

and the new combination of splitting functions by Ellis et al. (1996):

7 $$P_{ \pm } (x,\alpha ) = P_{V}^{(0)} + \frac{\alpha }{2\pi }P_{ \pm }^{(1)} (x) + \left( {\frac{\alpha }{2\pi }} \right)^{2} P_{ \pm }^{(2)} (x) + \cdots ,$$

the evolved DGLAP equations will be appeared in the following forms:

8 $$Q^{2} \frac{d}{{dQ^{2} }}q_{i}^{( - )} (x,Q^{2} ) = \frac{{\alpha (Q^{2} )}}{2\pi }P_{ - } (x,\alpha (Q^{2} )) \otimes q_{i}^{( - )} (x,Q^{2} ),$$

9 $$Q^{2} \frac{d}{{dQ^{2} }}\chi_{i} (x,Q^{2} ) = \frac{{\alpha (Q^{2} )}}{2\pi }P_{ + } (x,\alpha (Q^{2} )) \otimes \chi_{i} (x,Q^{2} ),$$

10 $$Q^{2} \frac{d}{{dQ^{2} }}\left( {\begin{array}{*{20}l} {q^{( + )} (x,Q^{2} )} \hfill \\ {G(x,Q^{2} )} \hfill \\ \end{array} } \right) = \frac{{\alpha (Q^{2} )}}{2\pi }\left( {\begin{array}{*{20}c} {P_{qq} (x,Q^{2} )} & \quad {P_{qg} {\mkern 1mu} (x,Q^{2} )} \\ {P_{gq} (x,Q^{2} )} & \quad {P_{gg} (x,Q^{2} )} \\ \end{array} } \right) \otimes \left( {\begin{array}{*{20}l} {q^{( + )} (x,Q^{2} )} \hfill \\ {G(x,Q^{2} )} \hfill \\ \end{array} } \right),$$

where ⊗ symbol is indicating the following convolution integral:

11 $$p(x) \otimes q(x) \equiv \int_{x}^{1} {\frac{dy}{y}p\left( {\frac{x}{y}} \right)q\left( y \right)} = \int_{x}^{1} {\frac{dy}{y}p(y)q\left( {\frac{x}{y}} \right)} .$$

Similar expansion like Eq. (7) exists for the different elements of the related matrix of splitting function. The advantage of using Eqs. (8–10) are that we are able to extract the sea quark densities at any energy scale *Q*^2^ separately for each quark flavor rather than to get an average quantity for sea quark densities.

Equations (8–10) can be written in terms of the new variable $t = - \frac{2}{{\beta_{0} }}\ln \frac{{\alpha (Q^{2} )}}{{\alpha (Q_{0}^{2} )}}\,\,$ so as:

12 $$\frac{d}{dt}q_{i}^{( - )} (x,t) = \left( {P_{V}^{(0)} (x) + \frac{\alpha }{2\pi }R_{ - } (x) + \cdots } \right) \otimes q_{i}^{( - )} (x,t),$$

13 $$\frac{d}{dt}\chi_{i} (x,t) = \left( {P_{V}^{(0)} (x) + \frac{\alpha }{2\pi }R_{ + } (x) + \cdots } \right) \otimes \chi_{i} (x,t),$$

14 $$\frac{d}{dt}\left( {\begin{array}{*{20}l} {q^{( + )} (x,t)} \hfill \\ {G(x,t)} \hfill \\ \end{array} } \right) = \left( {P^{(0)} (x) + \frac{\alpha }{2\pi }R(x) + \cdots } \right) \otimes \left( {\begin{array}{*{20}l} {q^{( + )} (x,t)} \hfill \\ {G(x,t)} \hfill \\ \end{array} } \right),$$

where

15 $$R_{ \pm } (x) = P_{ \pm }^{(1)} (x) - \frac{{\beta_{1} }}{{2\beta_{0} }}P_{V}^{(0)} (x),$$

16 $$R(x) = P^{(1)} (x) - \frac{{\beta_{1} }}{{2\beta_{0} }}P^{(0)} (x).$$

Solutions of Eqs. (10–12) will lead us to the evolved valence, sea and gluon densities at different energy scales.

### Evolution operators

Defining $\tilde{q}(x) \equiv q(t = 0,x)$ as parton density at initial energy scale *Q*_0_, the evolved valence density will be obtained by

17 $$q_{i}^{( - )} (t,x) = E_{ - } (t,x{\mkern 1mu} ) \otimes {\mkern 1mu} \tilde{q}_{i}^{( - )} (x).$$

For the *χ*_*i*_ function we will have

18 $$\chi_{i} (t,x) = E_{ + } (t,x{\mkern 1mu} ) \otimes {\mkern 1mu} \tilde{\chi }_{i} (x),$$

and for the gluon and singlet distributions, we will have:

19 $$\left( {\begin{array}{*{20}c} {q^{( + )} (t,x)} \\ {G(t,x)} \\ \end{array} } \right) = E(t,x) \otimes \left( {\begin{array}{*{20}c} {\tilde{q}^{( + )} (x)} \\ {\tilde{G}(x)} \\ \end{array} } \right).$$

In Eq. (19), the first term on the right hand side is the evolution operator which for the singlet and gluon densities has the following matrix form:

20 $$E(t,x) = \left( {\begin{array}{*{20}c} {E_{qq} (t,x)} &\quad {E_{qg} (t,x)} \\ {E_{gq} (t,x)} &\quad {E_{gg} (t,x)} \\ \end{array} } \right).$$

Substituting Eqs. (17–19) in Eqs. (12–14) will lead us to:

21 $$\frac{d}{dt}E_{ \pm } (t,x) = \left( {P_{V}^{(0)} (x) + \frac{\alpha }{2\pi }R_{ \pm } (x) + \cdots } \right) \otimes E_{ \pm } (t,x),$$

22 $$\frac{d}{dt}E(t,x) = \left( {P^{(0)} (x) + \frac{\alpha }{2\pi }R(x) + \cdots } \right) \otimes E(t,x).$$

We should note that evolution operators satisfy the following initial conditions

23 $$E_{ \pm } (0,x) = \delta (1 - x),\,\,\,\,\,\,E(0,x) = \delta (1 - x) \cdot I,$$

where *I* In Eq. (23) refers to the unit matrix with dimension 2.

### Laguerre expansion

The Laguerre polynomials can be represented by an alternative form as Arfken and Weber (2005):

24 $$L_{n} (x) = \sum\limits_{k = 0}^{n} {\left( {\begin{array}{*{20}c} n \\ k \\ \end{array} } \right)( - 1)^{k} \frac{{x^{k} }}{k!} = 1 - nx + \frac{n(n - 1)}{2!}\frac{{x^{2} }}{2!}} - \frac{n(n - 1)(n - 2)}{3!}\frac{{x^{3} }}{3!} + \cdots$$

These polynomials have the following properties:

1. The generating function of these polynomials is indicated by:
  25 $$g(x,z) = \frac{{e^{{{{ - xz} \mathord{\left/ {\vphantom {{ - xz} {\,(1 - z)}}} \right. \kern-0pt} {\,(1 - z)}}}} }}{1 - z} = \sum\limits_{n = 0}^{\infty } {L_{m} (x)z^{m} ,\quad \left| z \right| < 1} .$$
2. They satisfy the following recursive relation:
  26 $$L_{n + 1} (x) = 2L_{n} (x) - L_{n - 1} (x) - \frac{{(1 + x)L_{n} (x) - L_{n - 1} (x)}}{n + 1}.$$
3. These polynomials possess a closure property under the convolution integral:
  27 $$L_{n} (z) \otimes L_{m} (z) = L_{n + m} (z) - L_{n + m + 1} (z).$$
4. They also satisfy the following orthonormal condition, using the weight function *e*^−*y*^:
  28 $$\int_{0}^{\infty } {dye^{ - y} L_{m} (y)L_{n} (y) = \delta_{m,n} } .$$

Since the polynomials form a complete set, any function can be expanded in terms of them:

29 $$F(y) = \sum\limits_{m = 0}^{\infty } {F_{m} L_{m} (y)} ,$$

Following that we will have:

30 $$F_{m} = \int_{0}^{\infty } {dy\,e^{ - y} L_{m} (y)F(y)} .$$

Based on the completeness property, any two arbitrary functions *A*(*y*) and *B*(*y*) can be expanded in terms of Laguerre polynomials:

31 $$A(y) = \sum\limits_{n = 0}^{\infty } {A_{n} L_{n} (y)} ,\,\,\,\,\,B(y) = \sum\limits_{n = 0}^{\infty } {B_{n} L_{n} (y)} .$$

Assuming $C(y) = A(y) \otimes B(y) = \sum\nolimits_{n = 0}^{\infty } {C_{n} L_{n} (y)}$ and using the closure property, given by Eq. (27), the following relations can be obtained between the expansion coefficients

32 $$C_{n} = \sum\limits_{i = 0}^{n} {A_{i} b_{n - i} } = \sum\limits_{i = 0}^{n} {B_{i} a_{n - i} } ,$$

where

33 $$\begin{aligned} a_{i} = A_{i} - A_{i - 1} ,\,\,\,\,\,\,A_{ - 1} \equiv 0, \hfill \\ b_{i} = B_{i} - B_{i - 1} ,\,\,\,\,\,\,\,B_{ - 1} \equiv 0. \hfill \\ \end{aligned}$$

If we wish to use the Laguerre polynomials to get the evolved parton densities, we should change the interval of the *y* variable from (0, ∞) to (0, 1). Therefore, we need to change the variables as in the following

34 $$\begin{array}{*{20}l} {x = e^{ - y} ,} & \quad {dx = - xdy,} \hfill \\ {y:(0\;\infty ),} & \quad {x:(1\;0)\mathop{\longrightarrow}\limits{ - }(0\;1).} \hfill \\ \end{array}$$

Following that the orthonormal condition, given by Eq. (30), can be written as

35 $$\int_{0}^{1} {dxL_{m} \left( {\ln \frac{1}{x}} \right)L_{n} \left( {\ln \frac{1}{x}} \right) = \delta_{m,n} } .$$

Now the expansion of an arbitrary function *F*(*x*) would take the form

36 $$F(x) = \sum\limits_{n = 0}^{\infty } {F_{n} } L_{n} \left( {\ln \frac{1}{x}} \right),$$

so as for expansion coefficient, *F*_*n*_, we can write

37 $$F_{n} = \int_{0}^{1} d x{\mkern 1mu} L_{n} \left( {\ln \frac{1}{x}} \right)F(x).$$

Now we are equipped with the required relations to extract the evolution operators for parton densities in terms of the Laguerre polynomials which will be done in next subsection.

### Evolution operator for the non-singlet density: LO approximation

At the leading order (LO) approximation, Eq. (21) for the non-singlet evolution operator can be written as

38 $$\frac{d}{dt}E_{ - }^{(0)} (t,x) = P_{V}^{(0)} (x) \otimes E_{ - }^{(0)} (t,x),$$

Substituting Eqs. (32, 33) in Eq. (38), we arrive at

39 $$p_{i}^{(0)} = P_{i}^{(0)} - P_{i - 1}^{(0)} ,\,\,\,\,\,P_{ - 1}^{(0)} = 0,$$

40 $$\frac{d}{dt}\left( {E_{n}^{(0)} (t)} \right) = \sum\limits_{m = 0}^{n} {p_{n - m}^{(0)} E_{m}^{(0)} (t)} .$$

By using the initial condition, given by Eq. (23), for non-singlet sector, the general solution is

41 $$E_{n}^{(0)} (t) = e^{{P_{0}^{(0)} t}} \sum\limits_{k = 0}^{n} {\frac{{A_{n}^{(k)} t^{k} }}{{k{\mkern 1mu} !}}} ,$$

where

42 $$A_{n}^{(0)} = 1,\,\,\,\,\,\,\,A_{n}^{(k + 1)} = \sum\limits_{i = k}^{n - 1} {p_{n - i}^{(0)} } A_{i}^{(k)} .$$

By substituting Eq. (42) in Eq. (41), the evolution operator for the non-singlet sector at the LO approximation is determined and we can obtain parton densities at energy scale *Q*^2^ based on Eq. (17).

### Evolution operator for the non-singlet density: NLO approximation

We intend now to obtain the solution of the following differential equation

43 $$\frac{d}{dt}E_{ - } (t,x) = \left( {P_{V}^{(0)} (x) + \frac{\alpha }{2\pi }R_{ - } (x)} \right) \otimes E_{ - } (t,x),$$

where *R*_-_ is determined by Eq. (15). We can write the following Laguerre expansion for $R_{ - }$

44 $$R_{ - } (x) = \sum\limits_{n = 0}^{\infty } {R_{n} } L_{n} \left( {\ln \frac{1}{x}} \right).$$

Similar Laguerre expansions exist for $E_{ - } (t,x)$ which by substituting in Eq. (43), using Eqs. (32, 33) we will arrive at

45 $$\frac{d}{dt}\left( {E_{n} (t)} \right) = \sum\limits_{m = 0}^{n} {\left( {p_{n - m}^{(0)} + \frac{\alpha (t)}{2\pi }r_{n - m} } \right)} E_{m} (t),$$

where

46 $$r_{i} = R_{i} - R_{i - 1} ,\,\,\,\,\,R_{ - 1} = 0.$$

Now at the NLO approximation for *E*_*n*_(*t*), we can write

47 $$E_{n} (t) = E_{n}^{(0)} (t) + AE_{n}^{(1)} (t).$$

To simplify the calculation we present $AE_{n}^{(1)} (t)$ by *S*_*n*_(*t*). Therefore Eq. (45) can be written as

48 $$\frac{d}{dt}\left( {E_{n}^{(0)} (t)} \right) + \frac{d}{dt}\left( {S_{n} (t)} \right) = \sum\limits_{m = 0}^{n} {\left( {p_{n - m}^{(0)} + \frac{\alpha (t)}{2\pi }r_{n - m} } \right)} \left( {E_{m}^{(0)} (t) + S_{m} (t)} \right).$$

Using Eq. (40) and the initial condition, given by Eq. (23), we will get

49 $$S_{n} (t) = - \frac{{\beta_{0} }}{2}\frac{\alpha (t) - \alpha (0)}{2\pi }\sum\limits_{i = 0}^{n} {r_{n - i} } E_{i}^{(0)} (t).$$

In summary, the *E*_*n*_(*t*) term up to NLO approximation would have the following form

50 $$E_{n} (t) = E_{n}^{(0)} (t) - \frac{{\beta_{0} }}{2}\frac{\alpha (t) - \alpha (0)}{2\pi }E_{n}^{(1)} (t),$$

where

51 $$E_{n}^{(0)} (t) = e^{{P_{0}^{(0)} t}} \sum\limits_{k = 0}^{n} {\frac{{A_{n}^{(k)} t^{k} }}{{k{\mkern 1mu} !}}} ,\,\,\,\,\,\,\,\,\,\,E_{n}^{(1)} (t) = \sum\limits_{i = 0}^{n} {r_{n - i} E_{i}^{(0)} (t)} .$$

As in the LO approximation, by substituting Eq. (50) in the related expansion for $E_{ - } (t,x)$, the evolution operator for the non-singlet sector at the NLO approximation is determined and finally the parton densities at any energy scale can be obtained by using Eq. (17).

### Evolution operator for the singlet and gluon densities

This subsection contains two parts. At first, the solutions for the singlet sector, *q*^(+)^, and gluon densities are introduced. At the next step, using the solution for singlet sector and the *χ*_*i*_ distribution, it is possible to get the solution for $q_{i}^{( + )}$ which is defined by Eq. (5). Then, by accessing the valence distribution from the previous subsection and the $q_{i}^{( + )}$ distribution, sea quark distributions for individual flavors will be obtained. More details of these calculations are as followed.

In order to be able to extract sea quark densities, at first Eq. (9) should be solved and in terms of evolution operator, we will have [see Eq. (21)]:

52 $$\frac{d}{dt}E_{ + } (t,x) = \left( {P_{V}^{(0)} (x) + \frac{\alpha }{2\pi }R_{ + } (x) + \cdots } \right) \otimes E_{ + } (t,x).$$

The solution of this equation at the LO approximation is like the non-singlet case. At the NLO approximation, we should just do the following replacements with respect to the non-singlet case:

53 $$P_{ - } \to P_{ + } ,\,\,\,\,\,\,\,\,\,\,\,\,E_{ - } \to E,\,\,\,\,\,\,\,\,\,\,R_{ - } \to R_{ + } .$$

At the next step, Eq. (14) for singlet and gluon densities should be solved which at LO and NLO approximations could be done as follows:

#### LO approximation

At LO approximation, for related evolution operator, we have [see Eq. (22)]

54 $$\frac{d}{dt}\left( {\begin{array}{*{20}c} {E_{qq}^{(0)} (t,x)} &\quad {E_{qg}^{(0)} (t,x)} \\ {E_{gq}^{(0)} (t,x)} &\quad {E_{gg}^{(0)} (t,x)} \\ \end{array} } \right) = \left( {\begin{array}{*{20}c} {P_{qq}^{(0)} (x)} &\quad {P_{qg}^{(0)} (x)} \\ {P_{gq}^{(0)} (x)} &\quad {P_{gg}^{(0)} (x)} \\ \end{array} } \right) \otimes \left( {\begin{array}{*{20}c} {E_{qq}^{(0)} (t,x)} &\quad {E_{qg}^{(0)} (t,x)} \\ {E_{gq}^{(0)} (t,x)} &\quad {E_{gg}^{(0)} (t,x)} \\ \end{array} } \right).$$

The matrix evolution operator in Eq. (54) can be expanded in terms of Laguerre polynomials, so as

55 $$\left( {\begin{array}{*{20}c} {E_{qq}^{(0)} (t,x)} & \quad {E_{qg}^{(0)} (t,x)} \\ \quad {E_{gq}^{(0)} (t,x)} & \quad{E_{gg}^{(0)} (t,x)} \\ \end{array} } \right) = \sum\limits_{n = 0}^{\infty } {\left( {\begin{array}{*{20}c} {E_{n,qq}^{(0)} (t)} & \quad{E_{n,qg}^{(0)} (t)} \\ \quad {E_{n,gq}^{(0)} (t)} & \quad {E_{n,gg}^{(0)} (t)} \\ \end{array} } \right)L_{n} \left( {\ln \frac{1}{x}} \right)} .$$

The general solution for elements of the matrix evolution, by analytical consideration of moments, is as follows:

56 $$E^{(0)} (t,s) = e_{1} (s)\,e^{{\lambda_{1} (s)t}} + e_{2} (s)\,e^{{\lambda_{2} (s)t}} ,$$

where $e_{1} ,e_{2}$ are projection matrix operators with the following properties

57 $$e_{1} (s) + e_{2} (s) = I,\,\,\,\,\,\lambda_{1} (s)\,e_{1} (s)\, + \lambda_{2} (s)\,e_{2} (s)\, = \hat{P}^{(0)} (s),\,\,\,\,\,e_{i} (s)\,e_{j} (s) = \delta_{ij} \,e_{i} (s).$$

*λ*_1_(*s*) and *λ*_2_(*s*) are eigenvalues of the $\hat{P}^{(0)} (s)$ matrix which are given by

58 $$\lambda_{1} (1) \equiv \lambda = - \left( {\frac{4}{3}C_{F} + \frac{2}{3}n_{f} T_{R} } \right)$$

and due to momentum conservation at each vertex of parton splitting, we will have *λ*_2_(1) = 0 (Furmanski and Petroznio 1982a, b).

Considering the Laguerre expansion of evolution operator and also the splitting function in the Matrix form and Eq. (22) which connect these two expansions to each other, we will arrive at:

59 $$E_{n}^{(0)} (t) = \sum\limits_{k = 0}^{n} {\frac{{t^{k} }}{{k{\mkern 1mu} !}}} \left( {A_{n}^{(k)} + B_{n}^{(k)} e^{\lambda t} } \right).$$

In Eq. (59), the $A_{n}^{(k)}$ and $B_{n}^{(k)}$ coefficients are two dimensional matrix which are obtained from the following recurrence relations (Furmanski and Petroznio 1982a, b)

60 $$A_{0}^{(0)} = e_{2} ,\,\,\,\,\,\,\,\,\,\,B_{0}^{(0)} = e_{1} ,$$

61 $$\left\{ {\begin{array}{*{20}l} {A_{n}^{(k + 1)} = \lambda \,e_{1} \,A_{n}^{(k)} + \sum\nolimits_{i = k}^{n - 1} {p_{n - i}^{(0)} \,A_{i}^{(k)} ,} } \hfill & {n > 0,} \hfill \\ {B_{n}^{(k + 1)} = - \lambda \,e_{2} \,B_{n}^{(k)} + \sum\nolimits_{i = k}^{n - 1} {p_{n - i}^{(0)} \,B_{i}^{(k)} } ,} \hfill & {k = 0,1,2, \ldots ,n - 1.} \hfill \\ \end{array} } \right.$$

In deriving theses recurrence relations, Eq. (32) is used. Based on Eq. (57) the required matrix quantities in Eq. (61) are given by

62 $$\begin{aligned} & {e_{\,1} \equiv e_{\,1} (1),} \quad {e_{2} \equiv e_{\,2} (1),} \\ & {e_{1} = \frac{1}{\lambda }P_{0}^{(0)} ,} \quad {e_{2} = \frac{1}{\lambda }\left( { - P_{0}^{(0)} + \lambda I} \right) = - e_{1} + I.} \end{aligned}$$

At the beginning we need the initial values for $A_{n}^{(k)}$ and $B_{n}^{(k)}$ which are given by

63 $$\left\{ \begin{aligned} A_{n}^{(0)} = e_{2} - \frac{1}{{\lambda^{n} }}\left( {e_{1} \,a_{n}^{(n)} - ( - 1)^{n} e_{2} \,b_{n}^{(n)} } \right), \hfill \\ B_{n}^{(0)} = e_{1} + \frac{1}{{\lambda^{n} }}\left( {e_{1} \,a_{n}^{(n)} - ( - 1)^{n} e_{2} \,b_{n}^{(n)} } \right), \hfill \\ \end{aligned} \right.$$

where

64 $$\left\{ \begin{aligned} a_{n}^{(k + 1)} = \lambda \,e_{1} \,a_{n}^{(k)} + \sum\nolimits_{i = k}^{n - 1} {p_{n - i}^{(0)} \,A_{i}^{(k)} ,\,\,\,\,\,\,\,\,} a_{n}^{(0)} = 0, \hfill \\ b_{n}^{(k + 1)} = - \lambda \,e_{2} \,b_{n}^{(k)} + \sum\nolimits_{i = k}^{n - 1} {p_{n - i}^{(0)} \,B_{i}^{(k)} ,\,\,\,\,\,\,\,\,} b_{n}^{(0)} = 0. \hfill \\ \end{aligned} \right.$$

Substituting the coefficients $A_{n}^{(k)}$ and $B_{n}^{(k)}$ in Eq. (59) and the result of the related Laguerre expansion for the matrix evolution operator, the operator is obtained and we are able to use Eq. (19) to evolve singlet and gluon densities at higher energy scales. Equations (19, 20) at the LO approximation can be represented by:

65 $$\left\{ {\begin{array}{*{20}c} {q^{( + )} (t,x) = E_{qq}^{(0)} (t,x) \otimes \tilde{q}^{( + )} (x) + E_{qg}^{(0)} (t,x) \otimes \tilde{G}(x)} \\ {G(t,x) = E_{gq}^{(0)} (t,x) \otimes \tilde{q}^{( + )} (x) + E_{gg}^{(0)} (t,x) \otimes \tilde{G}(x)} \\ \end{array} } \right..$$

Using Eq. (65), we can obtain gluon and singlet distribution, *q*^(+)^, and then using the evolved valence quark (non-singlet) and *χ*_*i*_ distributions, Eqs. (17, 18), the sea distributions at the LO approximation will be obtained [see Eqs. (5, 6)]

66 $$\bar{q}_{i} = \frac{1}{2}\left( {\chi_{i} + \frac{1}{{n_{f} }}q^{( + )} - q_{i}^{V} } \right).$$

#### NLO approximation

The evolution operator in Eq. (22) at the NLO approximation is written as:

67 $$E(t,x) = E^{(0)} (t,x) + \frac{\alpha (t)}{2\pi }E^{(1)} (t,x).$$

The LO contribution, $E^{(0)} (t,x)$, is given by Eq. (59) and for the NLO contribution we should use the following relations (Furmanski and Petroznio 1982a, b)

68 $$E_{n}^{(1)} (t) = \tilde{E}_{n}^{(1)} (t) - 2\tilde{E}_{n - 1}^{(1)} (t) + \tilde{E}_{n - 2}^{(1)} (t),\quad \quad \tilde{E}_{ - 1}^{(1)} (t) = \tilde{E}_{ - 2}^{(1)} (t) = 0,$$

where $\tilde{E}_{n}^{(1)}$ can be obtained from $E_{n}^{(0)}$, using the following integral:

69 $$\tilde{E}_{n}^{(1)} (t) = \int_{0}^{t} {d\tau \,e^{{ - \beta_{0} \frac{\tau }{2}}} \sum\limits_{i,j,k} {E_{i}^{(0)} (t - \tau )\,R_{j} \,E_{k}^{(0)} (\tau )} \,\delta (n - i - j - k)} .$$

where $R_{j}$ is the expansion coefficients of *R*(*x*) in terms of Laguerre polynomials so as

$$R(x) = \sum\limits_{n = 0}^{\infty } {R_{n} \,} L_{n} \left( {\ln \frac{1}{x}} \right).$$

The sum in Eq. (69) should be used, considering the condition which is given by Dirac Delta function, that is *n* = *i* + *j* + *k*.

As before by accessing as well to the non-singlet and *χ*_*i*_ densities, using Eqs. (19, 50, 59, 68) the sea quark densities at the NLO approximation can be extracted

Now equipped by the required theoretical framework, based on Laguerre polynomial expansion, we will be able to get numerical results for the parton densities at any energy scale.

## Basic tools, Monte Carlo solutions

Here, we fully describe the numerical solutions of DGLAP evolution equations, using the FORTRAN programing language. At first, we introduce the general representations of programs, functions and subroutines which we are using in all our FORTRAN codes. The programs are divided into two parts, including the LO and NLO approximations; and each part contains a non-singlet and a singlet section. We then present the obtained numerical results by depicting the related parton densities at *Q*^2^ = 4, 50 and 200 GeV^2^. We also compare our results with the related results from CETQ and GRSV phenomenological groups. Comparisons with other numerical results have also been carried out.

### Functions, subroutines and main programs

We write the required codes in FORTRAN 90 language to solve numerically the DGLAP evolution equations. The basic method to solve the integrals is to use Monte Carlo simulations. The only generic subroutine used is *Ran3* which generates random numbers. All the other subroutines and function are written by us. We first introduce the functions and subroutines written by us and we will then illustrate the compiled programs in different sections.

#### Run3(idum) function

This function generates random numbers with uniform distribution between 0 and 1 based on Park-Miller method and *Knuth* suggested corrections. Each negative integer number can be considered as the input *idum*. The generation of this input should not be changed when we call them subsequently. The order of the period interval of this generation is 10^8^ (Press et al. 1996). The random numbers are generated in the interval [*a*, *b*], using *Ran3* function base on the following formula

70 $$y = a + (b - a)Ran\text{3}(idum).$$

Using Eq. (70) repeatedly, we can see that the generated numbers have uniform distribution and therefore we can use them to perform Monte Carlo integrations.

##### Monte Carlo integration

This method of integration is used to obtain the definite integrals which are based on generating random numbers. There are generally two different ways to do this kind of integration (Press et al. 1996).

I. Averaged Monte Carlo integration

For function, *f*(*x*) in the [*a*, *b*] interval, by getting the averaged function, $\bar{f}$, its integration can be obtained as follows:

71 $$\int_{a}^{b} {f(x)dx = } (b - a)\overline{f} .$$

To calculate the averaged function, we first generate N random number with uniform distribution in the [*a*, *b*] interval and then we get the averaged function, $\bar{f}$, according to

72 $$\bar{f}_{N} = \frac{{\sum\nolimits_{i = 1}^{N} {f(x_{i} )} }}{N}.$$

**Fig. 1:**
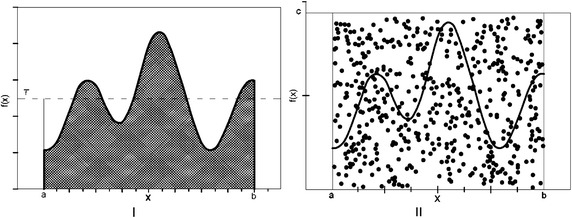
**I** An arbitrary function, f and its averaged value. **II** The generated pair number

Therefore, Eq. (71) can now be written as (see Fig. 1I)

73 $$\int_{a}^{b} {f(x)dx \approx } (b - a)\bar{f}_{N} .$$

II. Monte Carlo integrations, based on pair point method

**Fig. 2:**
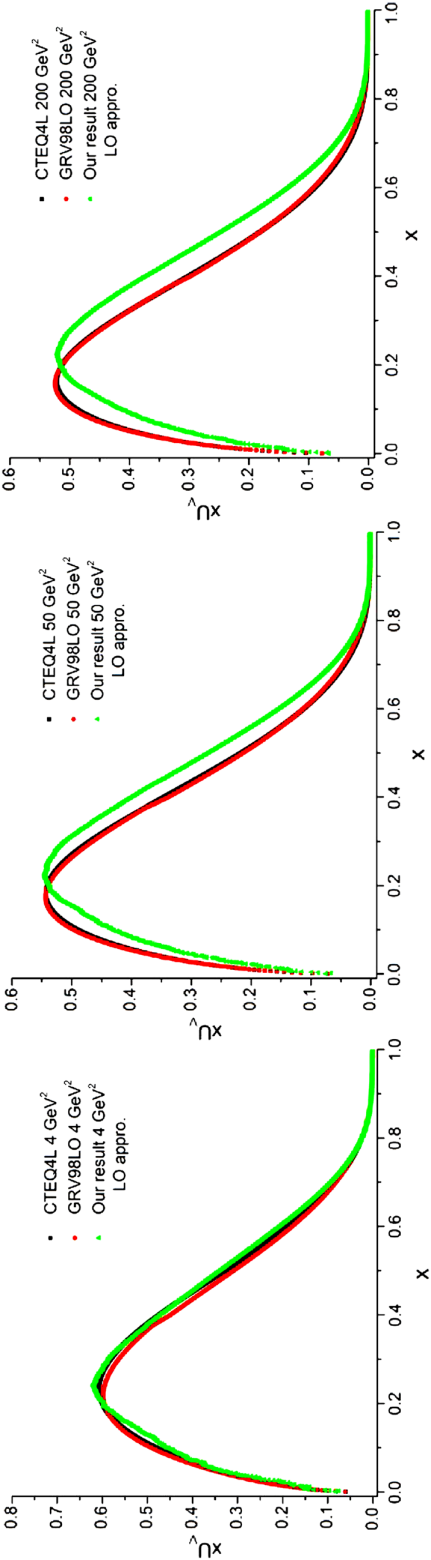
Valance u quark densities in the LO approximation at energy scales *Q* ^2^ = 4, 50, 200 GeV^2^. Comparison with the CETQ4L and GRSV98LO parameterization groups has also been done

In the first step, the random number,*x*_*i*_, is generated uniformly in the [*a*, *b*] interval [using Eq. (70)]. In the second step, the random number, *y*_*i*_, is generated in the [0, *c*] interval in which $c \ge f_{{\rm max} }$ and *f*_max_ is the maximum value of the function *f* in the [*a*, *b*] interval. Therefore, we achieve pairs of numbers (*x*_*i*_, *y*_*i*_) in a rectangle with *b* − *a* and *c* dimensions. By repeating the generation processes in the first and second steps N times, N pairs of numbers will be generated in the rectangle. According to Fig. 2II, if we count m pairs of numbers which are below *f*(*x*), we then have:

74 $$\int_{a}^{b} f(x)dx \approx \frac{m}{N}(b - a)c$$

We should note that for negative values of *y*_*i*_, we decrease one unit from m and consider the absolute value of the function, *f*, to produce the related pair. This method is more appropriate for huge complex integrals and by increasing the number of repeating processes, N, we get a better solution. After specified iterations, we get to a converged solution and we would not need to add to the number of repetitions.

#### Laguerre function, xlag(N,y,nmax)

The function *xlag*(*N,Y,nmax*) will give us numerical values for the Laguerre function at each order. By accessing the Laguerre function at two successive orders n and n − 1, the Laguerre function at the order n + 1 will be obtained using the following relation (Arfken and Weber 2005):

75 $$L_{n + 1} (x) = 2L_{n} (x) - L_{n - 1} (x) - \frac{{(1 + x)L_{n} (x) - L_{n - 1} (x)}}{n + 1}.$$

#### Subroutine intp0(p0,ymin,ymax,ndat,nmax)

This subroutine will produce the Laguerre expansion coefficient of the splitting function, using Monte Carlo integration. The input of this subroutine is the splitting function and the output is an array which contains the subtraction of subsequent expansion coefficients and is denoted by *p0*.

#### Subroutine intR(R,ymin,ymax,ndat,nmax)

This subroutine will produce the expansion coefficients of a combined splitting function at the NLO approximation in each order, using the Monte Carlo integration. The input of this subroutine is the splitting function and the output is an array which contains the subtraction of the subsequent expansion coefficients and is denoted by *rn.*

#### Splitting functions, FPn0(n,x,nmax),…

The outputs of these functions are the numerical values of splitting functions which are multiplied by Laguerre coefficient in which the existing singularities are removed by the plus prescription method. The plus prescription takes advantage the following relation (Greiner et al. 1996):

76 $$\int_{0}^{1} {dx\frac{f(x)}{{\left( {1 - x} \right)_{ + } }}} = \int_{0}^{1} {dx\frac{f(x) - f(1)}{1 - x}}$$

#### Function E0Lag(y0,ELO,nmax)

Considering the Laguerre expansion for the evolution operator which was introduced in subsection “Laguerre expansion”, the numerical values for this function can be obtained at each order of Laguerre expansion in terms of *t* and *x* variables.

#### Functions qinq(z), …

These functions are related to the parton densities at initial energy scale $Q_{0}^{2} = \,2.56\,{\text{GeV}}^{2}$ and their combinations which could be found in Lai et al. (1997). These functions are calculated using the generated random numbers, based on the Monte Carlo program. The results of two CTEQ4L and CETQ4M fitting groups are used to give us the initial parton densities at LO and NLO approximations respectively.

#### Function Zeta(is)

This function is defined by Ellis et al. (1996)

77 $$\zeta (s) = \sum\limits_{k = 1}^{\infty } {\frac{1}{{k^{s} }}} .$$

#### Function S2(Y)

The *S2*(*Y*) function is defined by Ellis et al. (1996)

78 $$S_{2} (x) = - 2Li_{2} ( - x) + \frac{1}{2}\ln^{2} (x) - 2\ln (x)\ln (1 + x) - \frac{{\pi^{2} }}{6}.$$

where *Li*_2_(*x*) as a dilogarithm function can be approximated by:

79 $$Li_{2} (x) = - \int_{0}^{x} {dy\frac{1 - y}{y}} = \frac{x}{{1^{2} }} + \frac{{x^{2} }}{{2^{2} }} + \frac{{x^{3} }}{{3^{2} }} + \cdots \quad for\,\left| x \right| \le 1.$$

The other required functions and subroutines will be introduced in the respective sections.

## The programs and the results

All programs are written in four parts:

### Non-singlet sector at the LO approximation

In this case, we ate concerned with the evolution of valence quarks. So according to notations of section “DGLAP evolution equations”, we just need to consider the DGLAP equation for $q_{i}^{( - )}$. To achieve this numerical solution, we do the following:

1. First, we should calculate numerically the expansion coefficients of splitting functions, using Eq. (37) where we choose the upper limit of summation in Eq. (36) equals to 30. To avoid the singularities which do exist in the splitting functions, we take into account the splitting function while we multiply the function with x rather than themselves and in the end, we display *x* times the parton densities. Therefore, the expansion coefficients are obtained via the following relation:
  80 $$P_{n}^{(0)} = \int_{0}^{1} d x{\mkern 1mu} L_{n} (\ln \left(\frac{1}{x}\right))\,xP_{V}^{(0)} (x),$$
  in which (Furmanski and Petronzio 1980):
  81 $$P_{V}^{(0)} (x) = P_{qq}^{(0)} (x) = C_{F} \left( {\frac{{1 + x^{2} }}{{\left( {1 - x} \right)_{ + } }} + \frac{3}{2}\delta (1 - x)} \right),$$
  The contribution of Dirac delta function in Eq. (81) is given by:
  82 $$\delta P_{n}^{(0)} = \int_{0}^{1} d x{\mkern 1mu} L_{n} (\ln \left(\frac{1}{x}\right))x\frac{3}{2}C_{F} \delta (1 - x) = \frac{3}{2}C_{F} = 2.$$
  Considering the rest of $P_{V}^{(0)} (x)$, the final result for Eq. (80) would be:
  83 $$P_{n}^{(0)} = C_{F} \int_{0}^{1} d x{\mkern 1mu} L_{n} \left( {\ln \left( {\frac{1}{x}} \right)} \right)x\frac{{1 + x^{2} }}{{\left( {1 - x} \right)_{ + } }} + \delta P_{n}^{(0)} .$$
  By applying the plus prescription,Eq. (76), the final result for $P_{n}^{(0)}$ is given by:
  84 $$P_{n}^{(0)} = C_{F} \int_{0}^{1} d x{\mkern 1mu} \left( {\frac{{L_{n} \left( {\ln \left( {\frac{1}{x}} \right)} \right)x(1 + x^{2} ) - 2}}{{\left( {1 - x} \right)}}} \right) + 2.$$
  The integral in Eq. (84) is done numerically, using the pair point method [see Eq. (74)]. The result of integral is given by *intp0* subroutine. The outputs of the program which contain the differences between two subsequent expansion coefficients will be saved in an array called *p0*(*0:nmax*) [see Eq. (39)].
2. Now using Eq. (42) it is possible to calculate numerically the matrix A. The sum which is relating to A matrix in Eq. (42) is calculated in *sumA*(*A,p0, nmax*) subroutine, where p0 is the output of *intp0* subroutine. Therefore A in *sumA*(*A,p0,nmax*) subroutine is a two dimensional array which is presented by $A(0:k\hbox{max} \,,\,0:n\hbox{max} )$ in our program.

Therefore, the sum in Eq. (42) can be calculated by doing three loops over I, k and n indices. The last index, I, contains by itself an additional sum. All these steps are performed in *sumA*(*A, p0, nmax*) subroutine. The general solution for the A matrix is as follows:

85 $$A = \left( {\begin{array}{*{20}c} {} &\quad {n = 0} &\quad {n = 1} &\quad {n = 2} &\quad . &\quad . &\quad . \\ {k = 0} &\quad 1 &\quad 1 &\quad 1 &\quad . &\quad . &\quad . \\ {k = 1} &\quad 0 &\quad {p_{1}^{(0)} } &\quad {p_{2}^{(0)} + p_{1}^{(0)} } &\quad . &\quad . &\quad . \\ {k = 2} &\quad 0 &\quad 0 &\quad {(p_{1}^{(0)} )^{2} } &\quad . &\quad . &\quad . \\ . &\quad . &\quad . &\quad . &\quad . &\quad . &\quad . \\ . &\quad . &\quad . &\quad . &\quad . &\quad . &\quad . \\ . &\quad . &\quad . &\quad . &\quad . &\quad . &\quad . \\ \end{array} } \right).$$

1. Now by considering the A matrix from step 2, and *p0* from the first step, it is possible to numerically calculate the evolution operator at LO approximation, using Eq. (41).

The related program is given by *ELOn*(*ELO,A,p0,nmax*) subroutine in which the variable *t*at the LO approximation has been defined:

86 $$t_{LO} = - \frac{2}{{\beta_{0} }}\ln \frac{{\alpha_{LO} (Q^{2} )}}{{\alpha_{LO} (Q_{0}^{2} )}},$$

where

87 $$\alpha_{LO} (Q^{2} ) = \frac{4\pi }{{\beta_{0} }}{\mkern 1mu} \frac{1}{{\ln \left( {\frac{{Q^{2} }}{{\varLambda^{2} }}} \right)}}.$$

The required quantities in the definition of *t* can be found in Gluck et al. (1998) [see Eq. (3)].

1. The convolution integral in Eq. (17) would now take the form:
  88 $$q_{i}^{( - )} (t,x) = E_{ - } (t,x{\mkern 1mu} ) \otimes {\mkern 1mu} \tilde{q}_{i}^{( - )} (x) = \int_{x}^{1} {E_{ - } \left( {t,\frac{x}{y}} \right)} \,{\mkern 1mu} \tilde{q}_{i}^{( - )} (y)\frac{dy}{y},$$
  or alternatively:
  89 $$q_{i}^{( - )} (t,x) = \int_{x}^{1} {E_{ - } \left( {t,\frac{x}{y}} \right)} {\mkern 1mu} \left[ {y\tilde{q}_{i}^{( - )} (y)} \right]\frac{dy}{{y^{2} }},$$
  since in the CETQ4 parameterization (Lai et al. 1997), the initial densities are presented as *x* times the parton densities. Considering the Laguerre expansion of evolution operator, Eq. (89) can be written as:
  90 $$q_{i}^{( - )} (t,x) = \int_{x}^{1} {\sum\limits_{n = 0}^{n\max } {E_{n}^{(0)} (t)L_{n} \left( {\frac{1}{{{x \mathord{\left/ {\vphantom {x y}} \right. \kern-0pt} y}}}} \right)} \left[ {y\tilde{q}_{i}^{( - )} (y)} \right]\frac{dy}{{y^{2} }}} .$$
  where $E_{n}^{(0)} (t)$ is given by Eq. (41). How to use it in the numerical calculation was described in previous step. In Eq. (90) $y\tilde{q}_{i}^{( - )} (y)$ represents the initial parton densities at energy scale $Q_{0}^{2} = \,2.56\,{\text{GeV}}^{2}$. The numerical solution of Eq. (90) is brought into the main part of program. For this propose we first need to perform a loop over *x* in the interval, say [0.001, 1] with the step 0.001 and then do the integration over *y* for each value of *x*.

**Fig. 3:**
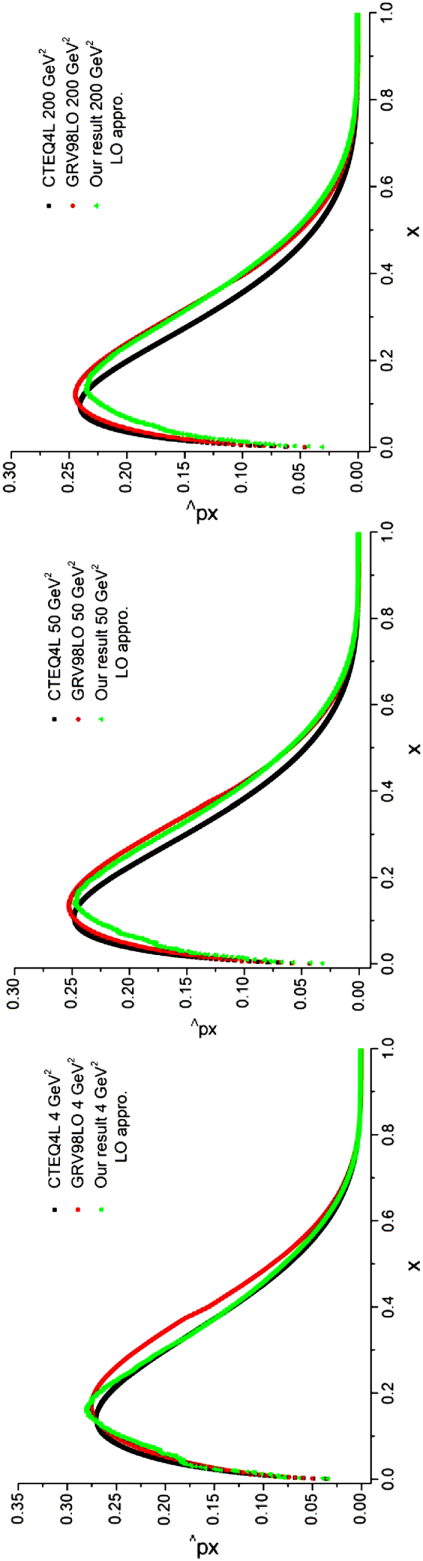
Valence d quark densities in the LO approximation at energy scales *Q* ^2^ = 4, 50, 200 GeV^2^ Comparison with the CETQ4L and GRSV98LO parameterization groups has also been done

We perform the integration using the pair point method [Eq. (74)] which is specified in the program by the index *pair.* This program is run at three energy scales *Q*^2^ = 4, 50, 200 GeV^2^ where the wall clock time depends on the produced random numbers. The output of the program is labeled “Our result”. The results are depicted in Figs. 2, 3 and compared with CTEQ (Lai et al. 1997) and GRV (Gluck et al. 1995) parameterization groups.

### Non-singlet sector at NLO approximation

In this case we should numerically solve Eq. (12) at the NLO approximation. We first need to calculate $R_{ - }$ which was defined by Eq. (15). This section, like the previous one, can be divided to four parts.

1. The required splitting functions at NLO approximation are could be found in Furmanski and Petronzio (1980), Herrod and Wada (1980). Whenever it is needed we use the plus prescription technique to remove the singularities in the calculations [see Eq. (76)]. To obtain the Laguerre expansion coefficients, all the splitting functions should be multiplied by $xL_{n} \left( {\ln 1/x} \right)$ and then we need to perform numerical integration as we did before [Eq. (80)]. It is required to separately perform the integration resulted from Dirac delta function in the splitting functions. These integrals together with the integrals whose singularities have been removed and also the rest of results, will produce the following functions which we need to run the program:
  $$xRnglag, \, xP0qqlag, \, xP1nglag, \, PF, \, PA, \, xPGlag, \, fPG, \, xPNFlag, \, fPNF.$$
  The subroutine which provides us with the Laguerre expansion coefficients of $P_{ - } \,,\,\,R_{ - }$ is called *intp0*(*p0,rn,xmin,xmax,ndat,mmax*). The contribution from Dirac delta function in the splitting function can be expressed by:
  91 $$\begin{aligned} \delta P_{n - }^{(1)} &= \int_{0}^{1} d x{\mkern 1mu} L_{n} \left( {\ln \left( {\frac{1}{x}} \right)} \right)x\left[ {C_{F}^{2} \delta (1 - x)\left( {\frac{3}{8} - \frac{1}{2}\pi^{2} + 6\zeta (3)} \right)} \right. \hfill \\ &\quad \left. + \frac{1}{2}C_{F} C_{A} \delta (1 - x)\left( {\frac{17}{12} + \frac{11}{9}\pi^{2} - 6\zeta (3)} \right) - C_{F} T_{R} n_{f} \delta (1 - x)\left( {\frac{1}{6} + \frac{2}{9}\pi^{2} } \right) \right] \hfill \\ &= C_{F}^{2} \left( {\frac{3}{8} - \frac{1}{2}\pi^{2} + 6\zeta (3)} \right) + \frac{1}{2}C_{F} C_{A} \left( {\frac{17}{12} + \frac{11}{9}\pi^{2} - 6\zeta (3)} \right) - C_{F} T_{R} n_{f} \left({\frac{1}{6} + \frac{2}{9}\pi^{2} } \right), \hfill \\ \end{aligned}$$
  92 $$\begin{aligned} \delta R_{n - } & = \delta P_{n - }^{1} (x) - \frac{{\beta_{1} }}{{2\beta_{0} }}\delta P_{n}^{(0)} (x) = C_{F}^{2} \left( {\frac{3}{8} - \frac{1}{2}\pi^{2} + 6\zeta (3)} \right) \\ & \quad + \frac{1}{2}C_{F} C_{A} \left( {\frac{17}{12} + \frac{11}{9}\pi^{2} - 6\zeta (3)} \right) - C_{F} T_{R} n_{f} \left( {\frac{1}{6} + \frac{2}{9}\pi^{2} } \right) - \frac{{\beta_{1} }}{{2\beta_{0} }}(2). \\ \end{aligned}$$
  The numerical values, resulted from Eqs. (91, 92) has been calculated in the *intp0* subroutine. In continue, by adding all the contributions from the splitting function, the related Laguerre expansion coefficients can be calculated as:
  93 $$R_{n - } = \int_{0}^{1} d x{\mkern 1mu} L_{n} \left( {\ln \left( {\frac{1}{x}} \right)} \right)xR_{ - } (x),$$
  94 $$R_{ - } (x) = \left( {P_{NS}^{(1)\, - } - \frac{{\beta_{1} }}{{2\beta_{0} }}P_{qq}^{(0)} (x)} \right),$$
  where $P_{NS}^{(1)\, - }$ has been introduced in Ref. Furmanski and Petronzio (1980), Herrod and Wada (1980).
  The numerical solutions of Eq. (93) are calculated in the *intp0* subroutine. The outputs of the subroutine are the differences between two subsequent coefficients of the expansion which will be put in *p0*(*0:nmax*) and *rn*(*0:nmax*) as two dimensional arrays.
2. Now by accessing the two arrays *p0*(*0:nmax*) and *rn*(*0:nmax)* it is possible to calculate the matrix A as before. The program does not need to be changed as we use *sumA*(*A,p0,nmax*) again and we simply change its inputs.
3. In this step, considering the matrix A which was made in the second step and the values of *p0* and *rn* which were calculated in the first step, it is possible to calculate the expansion coefficients of evolution operators, $E_{n}^{(0)} (t),\,\,E_{n}^{(1)} (t),\,\,E_{n} (t)$ [see Eqs. (50, 51)]. The related program is presented in *ENLOn*(*ENLO,A,p0,rn,nmax*) subroutine, as it follows: At first the variable:
  95 $$t_{NLO} = - \frac{2}{{\beta_{0} }}\ln \frac{{\alpha_{NLO} (Q^{2} )}}{{\alpha_{NLO} (Q_{0}^{2} )}},$$
  should be used where the coupling constant at NLO approximation is given by:
  96 $$\alpha_{NLO} (Q^{2} ) = \frac{4\pi }{{\beta_{0} }}{\mkern 1mu} \frac{1}{{\ln (\frac{{Q^{2} }}{{\varLambda^{2} }})}}\left( {1 - \frac{{\beta_{1} }}{{\beta_{0}^{2} }}\frac{{\ln \left( {\ln (\frac{{Q^{2} }}{{\varLambda^{2} }})} \right)}}{{\ln (\frac{{Q^{2} }}{{\varLambda^{2} }})}}} \right).$$
  The evolution operator at the NLO approximation in terms of the *t* variable is written as [see Eq. (50)]:
  97 $$E_{n} (t) = E_{n}^{(0)} (t) - \frac{{\beta_{0} }}{2}\frac{{\alpha (Q^{2} ) - \alpha (Q_{0}^{2} )}}{{(2\pi )^{2} }}E_{n}^{(1)} (t).$$

After performing all the numerical calculations in the mentioned subroutine, the output will be saved in a one dimensional array called *ENLO*.

1. This step is like step 4 of the previous part. The valence quark densities are obtained, using the following convolution integral:
  98 $$q_{i}^{( - )} (t,x) = \int_{x}^{1} {\sum\limits_{n = 0}^{n\max } {E_{n} (t)L_{n} \left( {\ln \frac{1}{{{x \mathord{\left/ {\vphantom {x y}} \right. \kern-0pt} y}}}} \right)} \left[ {y\tilde{q}_{i}^{( - )} (y)} \right]\frac{dy}{{y^{2} }}} .$$
  The expansion coefficients, *E*_*n*_(*t*), can be obtained via:
  99 $$E_{ - } (t,\frac{x}{y}) = \sum\limits_{n = 0}^{n\max } {E_{n} (t)} \,L_{n} \left( {\ln \frac{1}{{{x \mathord{\left/ {\vphantom {x y}} \right. \kern-0pt} y}}}} \right).$$
  Equation (99) is calculable with *E0lag* subroutine (see subsection “Function *E0Lag*(*y0,ELO,nmax*)”) where $E_{ - }$ is governed by Eq. (43) and since $R_{ - }$ and $P_{ - }$ are known, the expansion coefficients, *E*_*n*_(*t*), are calculable.

**Fig. 4:**
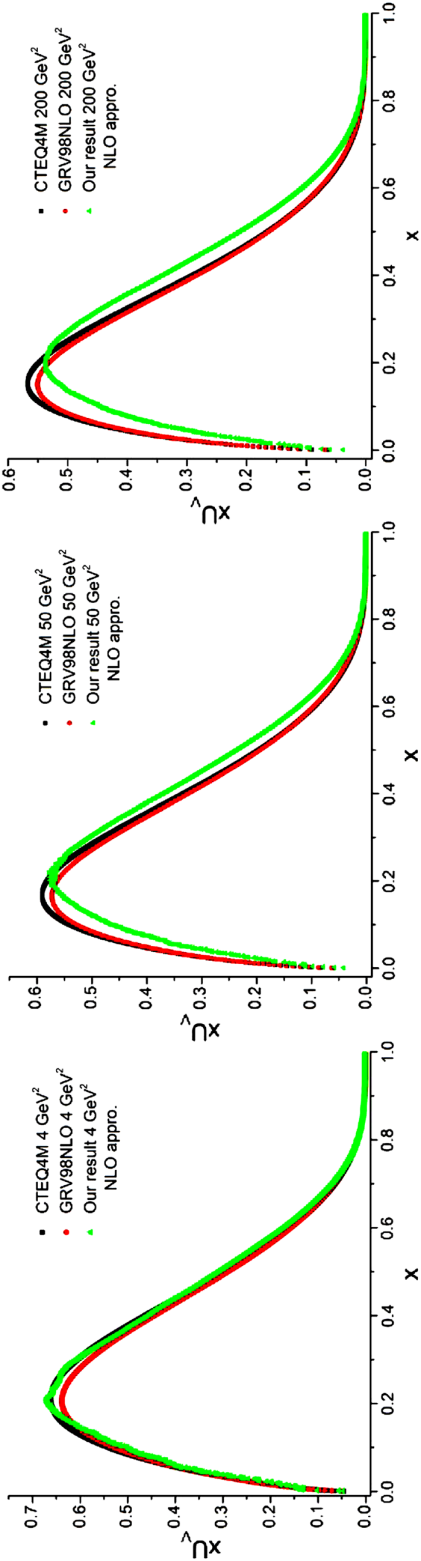
Valence u quark densities in the NLO approximation at energy scales *Q* ^2^ = 4, 50, 200 GeV^2^. Comparison with the CETQ4 M and GRSV98NLO parameterization groups has also been done

**Fig. 5:**
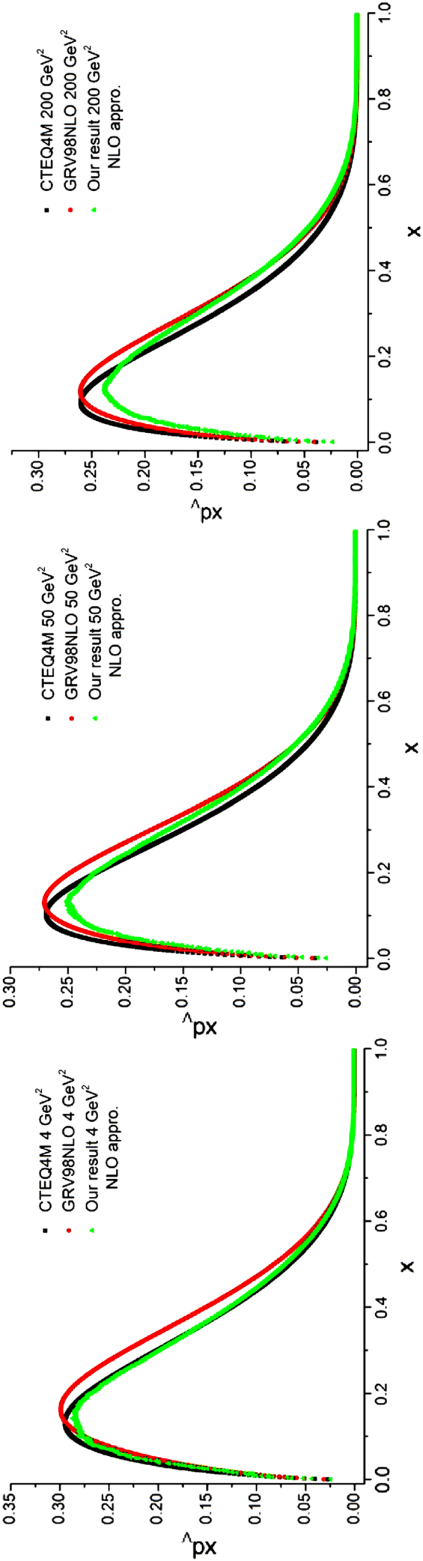
Valence d quark densities in the NLO approximation at energy scales *Q* ^2^ = 4, 50, 200 GeV^2^. Comparison with the CETQ4M and GRSV98NLO parameterization groups has also been done

To perform the integration in Eq. (98), we follow the previous method. The only difference is in the initial parton densities which would be the ones mentioned in Lai et al. (1997)) but at NLO approximation. The results of running the programs for valence *u* and *d* quark densities are depicted in Figs. 4, 5 and compared with the results from the CTEQ (Lai et al. 1997) and GRV (Gluck et al. 1995) parameterization groups.

To indicate the reliability of our numerical calculations, we should provide their statistical errors. This can be done, using the following relations:

99-a $$\int {f\,dx} \approx (x_{2} - x_{1} )\left\langle f \right\rangle \pm (x_{2} - x_{1} )\sqrt {\frac{{\left\langle {f^{2} } \right\rangle - \left\langle f \right\rangle^{2} }}{N}}$$

where “f” refers to related parton density. The last term in this equation indicated the error of calculations where 〈*f*^2^〉 and 〈*f*〉^2^ are given respectively by:

$$\left\langle f \right\rangle^{2} = \left( {\frac{1}{N}\sum\limits_{i = 1}^{N} {f(x_{i} )} } \right)^{2} \quad and \quad \left\langle {f^{2} } \right\rangle \quad = \left( {\frac{1}{N}\sum\limits_{i = 1}^{N} {f^{2} (x_{i} )} } \right).$$

In these relations N denotes to number of points where we used in the Mote Carlo numerical integration.

As a result what we got for statistical errors of different valance densities at typical value *Q*^2^ = 50 GeV^2^ and at the NLO approximation are as following:

$$xU_{v} \to \,\,\,\% 0.76\,,\,\,xD_{v} \,\, \to \,\,\% 0.33.$$

As can be seen these errors are <1 % which indicates enough reliability of our calculations for valence densities. The complete information for the other statistical errors at different energy scales for both LO and NLO approximations can be found in the appendix.

### Singlet sector at the leading order

In the singlet sector, we encounter functions which possess a matrix form. According to subsection “DGLAP evolution equations”, we should solve the Matrix evolution equation and also the equation for *χ*_*i*_, Eq. (14), in order to obtain the sea quark densities. First, the Gluon and *q*^(+)^ densities are obtained. Then, using the *q*^(+)^ and *χ*_*i*_, it is possible to get the *q*_*i*_^(+)^ densities. And finally, having the valence and $q_{i}^{( + )}$, the sea quark densities for any separate flavor is extractable.

For this section, two separate programs have been written and we first take into account the solution for the gluon density.

#### Gluon distribution at leading order

To get the solution for gluon densities, we should note that in almost all parts of the calculations we would encounter matrix form. This section involves three steps.

1. First, we need to define the splitting function (Furmanski and Petronzio 1980; Herrod and Wada 1980)
  100 $$P^{(0)} = \left( {\begin{array}{*{20}c} {P_{qq}^{(0)} } &\quad {P_{qg}^{(0)} } \\ {P_{gq}^{(0)} } &\quad {P_{gg}^{(0)} } \\ \end{array} } \right) \equiv \left( {\begin{array}{*{20}c} {P_{11}^{(0)} } &\quad {P_{12}^{(0)} } \\ {P_{21}^{(0)} } &\quad {P_{22}^{(0)} } \\ \end{array} } \right) \equiv P^{(0)} (IE,JE),\,\,\,\,\,\,\,\,IE,JE = 1,2.$$
  As can be seen, the splitting function is defined by a two dimensional array. As before the expansion coefficients of the splitting function should be determined which perform a three dimensional array:
  101 $$\begin{aligned} P_{n}^{(0)} &= \int_{0}^{1} d x{\mkern 1mu} L_{n} (\ln \left(\frac{1}{x}\right))xP^{(0)} (x), \hfill \\ P_{n}^{(0)}& \equiv P^{(0)} (n,IE,JE). \hfill \\ \end{aligned}$$

Equation (101) is in fact a matrix form which should be applied for all the elements in Eq. (100). It is obvious that we need to resort to the plus prescription technique to overcome the singularities of integration in Eq. (101). The concerned integration has been solved by the first method of numerical integration which is called the “averaged method” [see Eq. (73)]. The numerical solution of the related integral is inserted in the *intp0*(*p0,e1,e2,xmin,xmax,ndat,nmax*) subroutine. The output of this subroutine is the difference between the two subsequent expansion coefficients which is put in a one dimensional array, named *p0*(*0:nmax*).

The projection operators *e*_1_ and *e*_2_ are finally given by Eq. (62):

102 $$e_{1} = \frac{1}{\lambda }P^{(0)} (0,IE,JE) \equiv e1(IE,JE),\,\,\,\,\,\,e_{2} = - e_{1} + I\, \equiv e2(IE,JE).$$

2. Secondly, we should computed the A and B matrices, given by Eq. (61). To define the related arrays, we first consider the upper index (k) and then the lower index (n) and then we proceed to the indices of the 2 × 2 projection matrices which are represented by *IE*, *JE* symbols. So A and B construct 4 × 4 dimensional arrays which can be represented by:
  103 $$\begin{aligned} A(0:k\hbox{max} \,,\,0:n\hbox{max} ,2,2) \to A(k,\,n,IE,JE), \hfill \\ B(0:k\hbox{max} \,,\,0:n\hbox{max} ,2,2) \to B(k,\,n,IE,JE). \hfill \\ \end{aligned}$$
  The limit of the arrays are specified in the left hand side of Eq. (103). The *IE*, *JE* symbol is represented by 2 × 2 matrices.

Equations (61, 63, 64) are three recurrence relationships. To get the related solutions, we first need to calculate them for *n* = 0. Therefore we will have:

104 $$\left\{ \begin{aligned} a_{n}^{(0)} = 0\,\,\, \Rightarrow \,\,\,a_{0}^{(0)} = 0, \hfill \\ b_{n}^{(0)} = 0\,\,\, \Rightarrow \,\,\,b_{0}^{(0)} = 0, \hfill \\ \end{aligned} \right.$$

105 $$\left\{ \begin{aligned} a_{n}^{(k)} = b_{n}^{(k)} = 0,\,\,\,k \ge n \hfill \\ A_{n}^{(k)} = B_{n}^{(k)} = 0,\,\,\,k > n \hfill \\ \end{aligned} \right.$$

106 $$A_{0}^{(0)} = e_{2} ,\,\,\,\,\,B_{0}^{(0)} = e_{1} ,$$

These are considered as the required constants in our calculations. To proceed, we would consider *n* = 1; therefore, we would have

107 $$\mathop{\longrightarrow}\limits^{n = 1}\left\{ \begin{aligned} a_{1}^{(k + 1)} = \lambda \,e_{1} \,a_{1}^{(k)} + \sum\limits_{i = k}^{0} {p_{1 - i}^{(0)} \,A_{i}^{(k)} \mathop{\longrightarrow}\limits^{k = 0}\,a_{1}^{(1)} = p_{1}^{(0)} \,A_{0}^{(0)} = p_{1}^{(0)} e_{2} } \hfill \\ b_{1}^{(k + 1)} = - \lambda \,e_{2} \,b_{1}^{(k)} + \sum\limits_{i = k}^{0} {p_{1 - i}^{(0)} \,B_{i}^{(k)} \mathop{\longrightarrow}\limits^{k = 0}\,b_{1}^{(1)} = p_{1}^{(0)} \,B_{0}^{(0)} = p_{1}^{(0)} e_{1} } \hfill \\ \end{aligned} \right.,$$

108 $$\mathop{\longrightarrow}\limits{n = 1}\left\{ \begin{aligned} A_{1}^{(0)} = e_{2} - \frac{1}{{\lambda^{1} }}\left( {e_{1} \,a_{1}^{(1)} - ( - 1)^{1} e_{2} \,b_{1}^{(1)} } \right) \Rightarrow A_{1}^{(0)} = e_{2} - \frac{1}{\lambda }\left( {e_{1} p_{1}^{(0)} e_{2} \, + e_{2} p_{1}^{(0)} e_{1} } \right) \hfill \\ B_{1}^{(0)} = e_{1} + \frac{1}{{\lambda^{1} }}\left( {e_{1} \,a_{1}^{(1)} - ( - 1)^{1} e_{2} \,b_{1}^{(1)} } \right) \Rightarrow B_{1}^{(0)} = e_{1} + \frac{1}{\lambda }\left( {e_{1} p_{1}^{(0)} e_{2} \, + e_{2} p_{1}^{(0)} e_{1} } \right) \hfill \\ \end{aligned} \right.,$$

109 $$\mathop{\longrightarrow}\limits^{n = 1}\left\{ \begin{aligned} A_{1}^{(k + 1)} = \lambda \,e_{1} \,A_{1}^{(k)} + \sum\limits_{i = k}^{0} {p_{1 - i}^{(0)} \,A_{i}^{(k)} \mathop{\longrightarrow}\limits^{k = 0}A_{1}^{(1)} = \lambda \,e_{1} \,A_{1}^{(0)} + p_{1}^{(0)} e_{2} } \hfill \\ B_{1}^{(k + 1)} = - \lambda \,e_{2} \,B_{1}^{(k)} + \sum\limits_{i = k}^{0} {p_{1 - i}^{(0)} \,B_{i}^{(k)} \mathop{\longrightarrow}\limits^{k = 0}B_{1}^{(1)} = - \lambda \,e_{2} \,B_{1}^{(0)} + p_{1}^{(0)} \,e_{1} } \hfill \\ \end{aligned} \right..$$

The above relations can be extended to *n* > 1. So we can acquire all the required values of A and B matrices. There are four A and B matrices which are given in the program by *IE, JE*(qq, qg, gq, gg). The general forms of the matrices are:

110 $$A,B\left( {k,n} \right) = 0\,,\,\,\,\,\,for\,\,\,\,\,\,k > n.$$

All the calculation are performed in the *ABELO*(*E0,p0,e1,e2,nmax*) subroutine. The inputs of this subroutine are *e*_1_, *e*_2_ and *p*0 matrices and the outputs are the Laguerre expansion coefficients of the evolution operator, Eq. (59), at LO approximation, where they are put in the three dimensional array *E0*(*n,IE,JE*).

3. By achieving the Laguerre expansion coefficients, the evolution matrix operator in the Bjorken *x* space is calculable. Equation (55) is given by *E0Lag* function which which is called in the main program whenever is needed. We should note that at LO approximation, *E*^±^ are equal to*E*^(0)^. The combinations which give us the required densities are as follow:
  111 $$q_{i}^{( + )} = q_{i} + \bar{q}_{i},\quad q_{i}^{( - )} = q_{i}^{V} = q_{i} - \bar{q}_{i} \,\,\,\,\, \Rightarrow \,\,\,\,\,q_{i}^{( + )} = q_{i}^{V} + 2\,\bar{q}_{i} ,$$
  112 $$q^{( + )} = \Sigma {\mkern 1mu} = \sum\limits_{i = 1}^{{n_{f} }} {q_{i}^{( + )} } = \sum\limits_{i = 1}^{{n_{f} }} {q_{i}^{V} + 2\,\bar{q}_{i} } = q_{u}^{V} + 2\,\bar{q}_{u} + q_{d}^{V} + 2\,\bar{q}_{d} + 2\,\bar{q}_{s} .$$

As before, the parton densities at initial energy scale $Q_{0}^{2} = \,2.56\,\hbox{GeV}^{2}$ are taken form (Lai et al. 1997). The used density functions in the program are given by:

$$qinq, \, qinU, \, qind, \, qinqUb, \, qindb, \, qinSb, \, qinG$$

The first function is related to *q*^(+)^. The second and the third ones are related to valence quarks. The rest are related to sea quarks except the last one which is for the gluon density.

113 $$x\tilde{\bar{d}} = \frac{1}{2}\left( {x(\tilde{\bar{d}} + \tilde{\bar{u}})\, + x(\tilde{\bar{d}} - \tilde{\bar{u}})} \right),$$

114 $$x\tilde{\bar{u}} = \frac{1}{2}\left( {x(\tilde{\bar{d}} + \tilde{\bar{u}})\, - x(\tilde{\bar{d}} - \tilde{\bar{u}})} \right),$$

115 $$\tilde{q}^{( + )} = \tilde{u}_{v} + \tilde{d}_{v} + 2\,(\tilde{\bar{u}} + \tilde{\bar{d}} + \tilde{\bar{s}}).$$

The inputs of these functions are the generated random numbers by Monte Carlo method which produce numerical values for the related parton densities at initial energy scales. Since the density functions are appeared as *xq*, for $\tilde{q}^{( + )}$ we will have:

116 $$x\tilde{q}^{( + )} = x\tilde{q}_{u}^{V} + x\tilde{q}_{d}^{V} + 2\,(x\tilde{\bar{q}}_{u} + x\tilde{\bar{q}}_{d} + x\tilde{\bar{q}}_{s} )$$

Now, using the input density functions, the convolution integrals, Eq. (65) and the function *E0Lag* which is related to Eq. (55), it is possible to get the $q^{( + )}$ and gluon densities as in the following:

117 $$q^{( + )} (t,x) = \int_{x}^{1} {\left\{ {\sum\limits_{n = 0}^{n{\text{max}} } {E_{n,qq}^{(0)} (t)L_{n} \left( {\ln \frac{y}{x}} \right)} \left[ {y\tilde{q}^{( + )} (y)} \right] + \sum\limits_{n = 0}^{n{\text{max}} } {E_{n,qg}^{(0)} (t)L_{n} \left( {\ln \frac{y}{x}} \right)} \left[ {y\tilde{G}(y)} \right]} \right\}} \frac{dy}{{y^{2} }},$$

118 $$G(t,x) = \int_{x}^{1} {\left\{ {\sum\limits_{n = 0}^{n{\text{max}}} {E_{n,gq}^{(0)} (t)L_{n} \left( {\ln \frac{y}{x}} \right)} \left[ {y\tilde{q}^{( + )} (y)} \right] + \sum\limits_{n = 0}^{n{\text{max}} } {E_{n,gg}^{(0)} (t)L_{n} \left( {\ln \frac{y}{x}} \right)} \left[ {y\tilde{G}(y)} \right]} \right\}} \frac{dy}{{y^{2} }}.$$

**Fig. 6:**
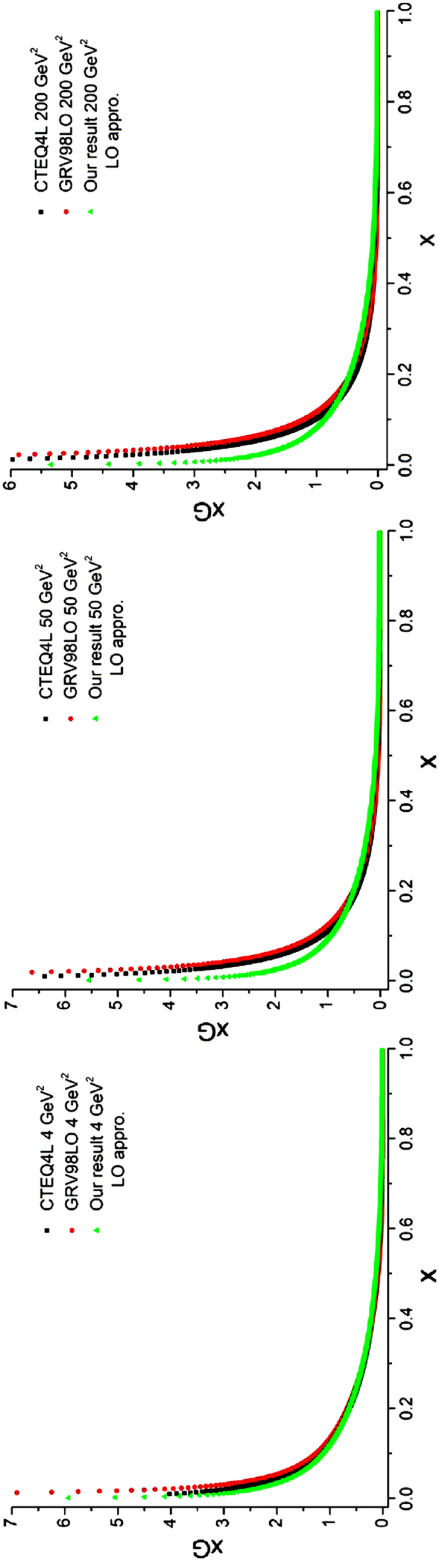
Gluon densities in the LO approximation at energy scales *Q* ^2^ = 4, 50, 200 GeV^2^. Comparison with CETQ4L and GRSV98LO parameterization groups has also been done

The integrals in Eqs. (117, 118) are obtained numerically based on the “average method” [see Eq. (73)]. The above integrals are used in the main part of the program. The results include *q*^(+)^ and gluon densities. We would require *q*^(+)^ in further sections to obtain the sea quark densities. The results of running the programs for gluon densities at different energy scales are depicted in Fig. 6 compared to the results from the CTEQ (Lai et al. 1997) and GRV (Gluck et al. 1995) parameterization groups.

#### Sea quark densities at LO approximation

This section contains two parts and for each part we require a separate program. At first, the equation which is related to *χ* distribution is solved. Then using the valence (obtained in 4.2) and *q*^(+)^ densities which were obtained in subsection “Gluon distribution at leading order”, we would be able to extract sea and gluon densities. This subsection is divided to two steps:

1. At this step we should first solve Eq. (9) for *χ*_*i*_ distribution. The related solution is like the one for the non-singlet distribution (subsection “Non-singlet sector at NLO approximation”) except that the replacement given by Eq. (53) should be carried out.
  Therefore we will have [see Eq. (13)]
  119 $$\frac{d}{dt}\chi_{i} (t,x) = \left( {P_{qq}^{(0)} (x) + \frac{\alpha }{2\pi }R_{\, + } (x)} \right) \otimes \chi_{i} (t,x),$$
  where
  120 $$R_{\, + } (x) = \left( {P_{NS}^{(1) + } - \frac{{\beta_{1} }}{{2\beta_{0} }}P_{qq}^{(0)} (x)} \right).$$

The analytical form for $P_{NS}^{(1) + }$ can be found in Furmanski and Petronzio (1980), Herrod and Wada (1980). In the written program we need to define two functions (*xRpolag*, *xP1polag*) which should be replaced by previous ones in subsection “Non-singlet sector at NLO approximation” (*xRnglag*, *xP1nglag*). The other stages, are just the same as the ones in section “Non-singlet sector at NLO approximation”. Since we have used *R*_+_ and *P*_+_, the obtained coefficients are *E*_+_. And after that, it would be possible to obtain the *χ*_*i*_(*x*, *t*) distribution in Bjorken *x*-space by Eq. (18).

The initial $\tilde{\chi }_{i}$ densities for separated quark flavor will be obtained, using the Eq. (66) as follows:

121 $$\left\{ {\begin{array}{*{20}l} {x\tilde{\chi }_{u} = x\tilde{u}_{v} + 2x\tilde{\bar{u}} - \frac{1}{{n_{f} }}x\tilde{q}^{( + )} ,} \hfill \\ {x\tilde{\chi }_{d} = x\tilde{d}_{v} + 2x\tilde{\bar{d}} - \frac{1}{{n_{f} }}x\tilde{q}^{( + )} ,} \hfill \\ {x\tilde{\chi }_{s} = 2x\tilde{\bar{s}} - \frac{1}{{n_{f} }}x\tilde{q}^{( + )} .} \hfill \\ \end{array} } \right.$$

The above functions are added to the program by the following names:

$$xkhiU, \, xkhid, \, xkhiS, \, qinq, \, qinU, \, qind, \, qinU, \, qinUb, \, qindb, \, qinSb, \, qinG \, .$$

Having accesses to the initial parton densities from Lai et al. (1997), which are recognized by the tilde symbol, we will get the *χ*_*i*_ densities for separate flavor as in the following:

122 $$\left\{ {\begin{array}{*{20}l} {\chi_{u} (t,x) = \int_{x}^{1} {E_{ + } (t,\frac{x}{y})\left[ {y\tilde{u}_{v} + 2y\tilde{\bar{u}} - \frac{1}{{n_{f} }}y\tilde{q}^{( + )} } \right]} \frac{dy}{{y^{2} }},} \hfill \\ {\chi_{d} (t,x) = \int_{x}^{1} {E_{ + } (t,\frac{x}{y})\left[ {y\tilde{d}_{v} + 2y\tilde{\bar{d}} - \frac{1}{{n_{f} }}y\tilde{q}^{( + )} } \right]} \frac{dy}{{y^{2} }},} \hfill \\ {\chi_{s} (t,x) = \int_{x}^{1} {E_{ + } (t,\frac{x}{y})\left[ {2y\tilde{\bar{s}} - \frac{1}{{n_{f} }}y\tilde{q}^{( + )} } \right]} \frac{dy}{{y^{2} }}.} \hfill \\ \end{array} } \right.$$

The integrals in Eq. (122) can be solved numerically, using the “average method” [see Eq. (73)]. The results would be the *χ*_*i*_ distributions at different energy scales.

2. It is now possible to get the sea quark densities at different energy scales, using Eq. (66):
  123 $$x\bar{u} = \frac{1}{2}\left( {x\chi_{u} + \frac{1}{{n_{f} }}xq^{( + )} - xu_{v} } \right),$$
  124 $$x\bar{d} = \frac{1}{2}\left( {x\chi_{d} + \frac{1}{{n_{f} }}xq^{( + )} - xd_{v} } \right),$$
  125 $$x\bar{s} = \frac{1}{2}\left( {x\chi_{s} + \frac{1}{{n_{f} }}xq^{( + )} } \right).$$

**Fig. 7:**
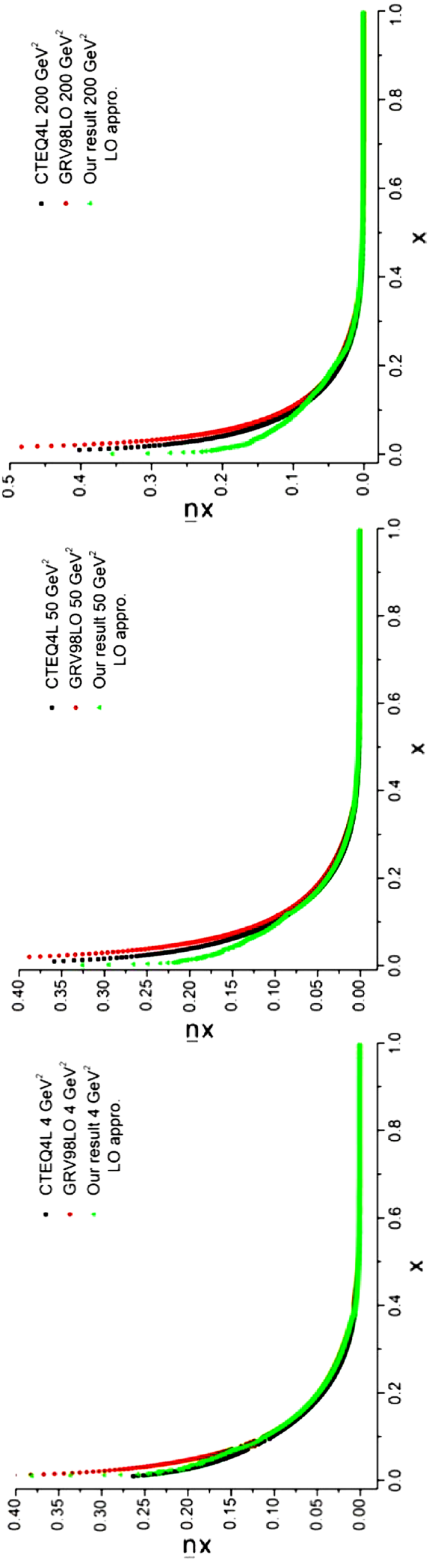
Sea u quark densities in the LO approximation at energy scales *Q* ^2^ = 4, 50, 200 GeV^2^. Comparison with CETQ4L and GRSV98LO parameterization groups has also been done

**Fig. 8:**
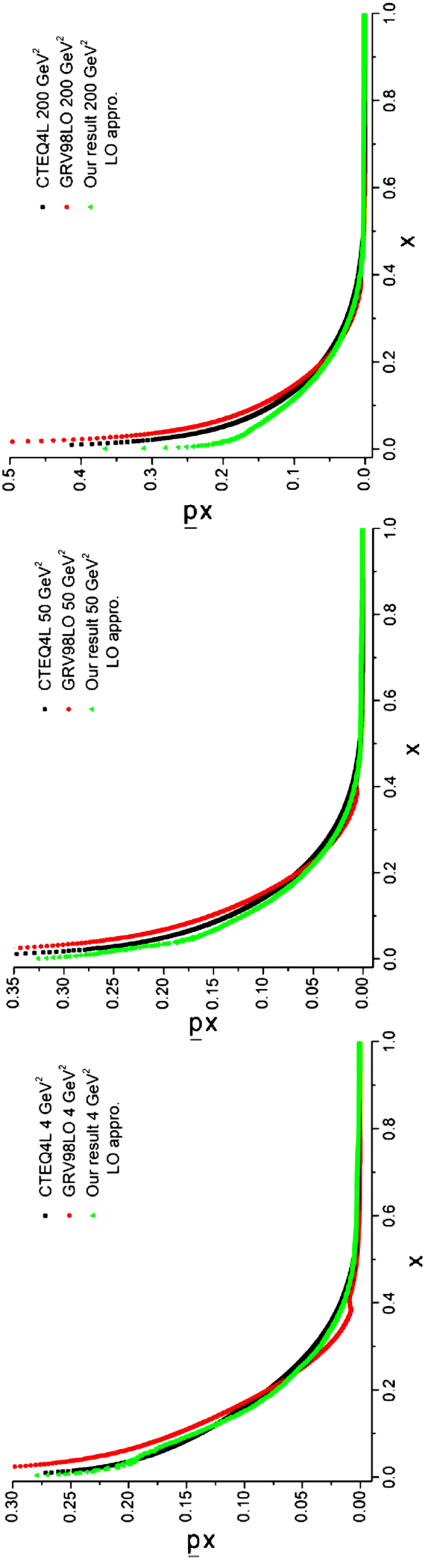
Sea d quark densities in the LO approximation at energy scales *Q* ^2^ = 4, 50, 200 GeV^2^. Comparison with CETQ4L and GRSV98LO parameterization groups has also been done

**Fig. 9:**
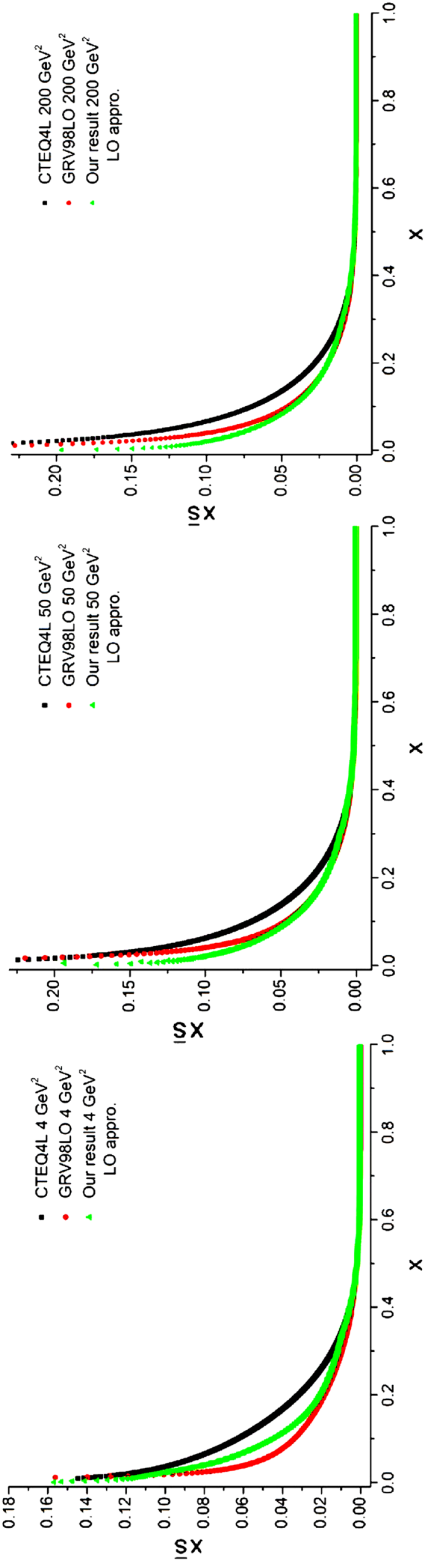
Sea s quark densities in the LO approximation at energy scales *Q* ^2^ = 4, 50, 200 GeV^2^. Comparison with CETQ4L and GRSV98LO parameterization groups has also been done

The required densities in Eqs. (123–125), including the valence (subsection “Non-singlet sector at NLO approximation”), *χ*_*i*_ [Eq. (122)] and *q*^(+)^ (subsection “Gluon distribution at leading order”) distributions have been obtained before. The results of running the programs for sea quark densities at energy scales, *Q*^2^ = 4, 50, 200 GeV^2^ are depicted in Figs. 7, 8 and 9 and compared with the results from CTEQ (Lai et al. 1997) and GRV (Gluck et al. 1995) parameterization groups.

### Singlet sector at the next leading order

As before this section includes two subsections:

#### Gluon densities at NLO approximation

The required Laguerre expansion coefficients for the evolution operator is given by Eq. (97). The $E_{n}^{(1)} (t)$ term in Eq. (97) is defined by Eqs. (68, 69). The only quantity in Eq. (69) which should be determined is *R*_*j*_. In the following, the gluon at NLO approximation can be calculated based on the following steps.

**Fig. 10:**
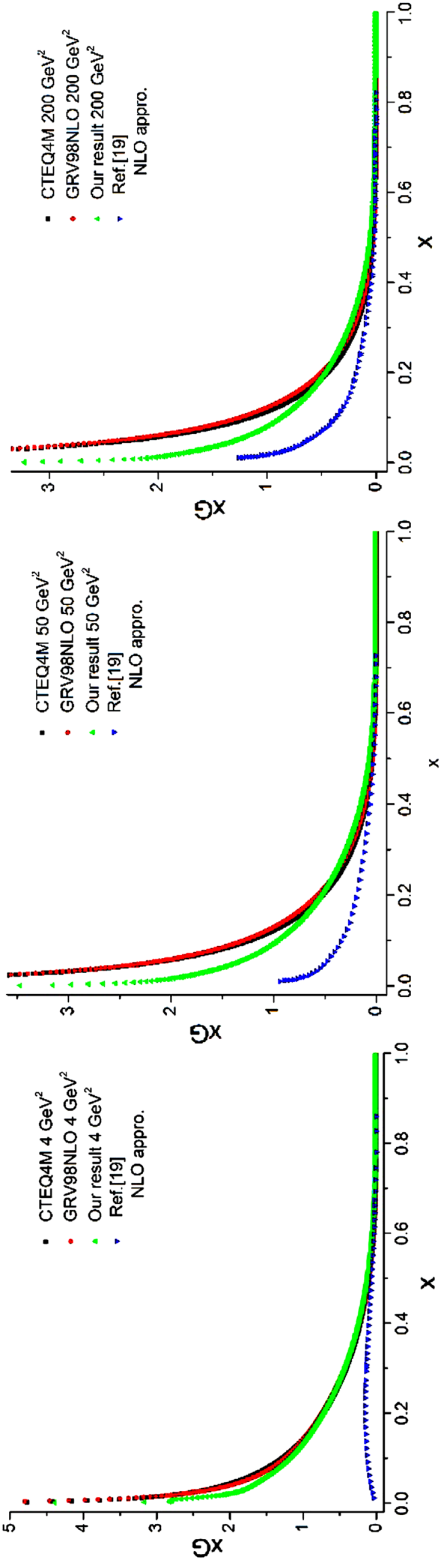
Gluon densities in the NLO approximation at energy scales *Q* ^2^ = 4, 50, 200 GeV^2^. Comparison with CETQ4M and GRSV98NLO parameterization groups and the results from Ref. Coriano and Savkli (1999) has also been done. Our results for gluon densities are in better agreement with the CETQ4M and GRSV98NLO results rather than the results from Ref. Coriano and Savkli (1999)

1. The calculation of *R*_*j*_ is done by a subroutine called *intR*(*R,xmin,xmax,ndat,nmax*). Evolution of gluon densities is done by Eq. (14) where *P*^(1)^(*x*) and *R*(*x*) in Eq. (16) are defined by:
  126 $$P^{(1)} = \left( {\begin{array}{*{20}c} {P_{qq}^{(1)} } & {P_{qg}^{(1)} } \\ {P_{gq}^{(1)} } & {P_{gg}^{(1)} } \\ \end{array} } \right) \equiv \left( {\begin{array}{*{20}c} {P_{11}^{(1)} } & {P_{12}^{(1)} } \\ {P_{21}^{(1)} } & {P_{22}^{(1)} } \\ \end{array} } \right) \equiv P^{(1)} (IE,JE),\,\,\,\,\,\,\,\,IE,JE = 1,2$$
  127 $$\left( {\begin{array}{*{20}c} {R_{qq} } &\quad {R_{qg} } \\ {R_{gq} } &\quad {R_{gg} } \\ \end{array} } \right) = \left( {\begin{array}{*{20}c} {P_{qq}^{(1)} } &\quad {P_{qg}^{(1)} } \\ {P_{gq}^{(1)} } &\quad {P_{gg}^{(1)} } \\ \end{array} } \right) - \frac{{\beta_{1} }}{{2\beta_{0} }}\left( {\begin{array}{*{20}c} {P_{qq}^{(0)} } &\quad {P_{qg}^{(0)} } \\ {P_{gq}^{(0)} } &\quad {P_{gg}^{(0)} } \\ \end{array} } \right),$$
  while matrix form for *P*^(0)^(*x*) is given by Eq. (100). As before, these matrices are defined by two dimensional arrays. The analytical expression for the splitting function at NLO approximation can be found in Furmanski and Petronzio (1980), Herrod and Wada (1980). The used functions in the program whose singularities have been removed by plus prescription technique [Eq. (76)] are:
  $$\begin{aligned} & xRqqlag, \, xRqglag, \, xRgqlag, \, xRgglag, \, xP0qqlag, \, xP0qglag, \, xP0gqlag, \, xP0gglag, \, xP1qqlag, \hfill \\ & xP1qglag, \, xP1gqlag, \, xP1gglag, \, xFqqlag, \, xF1qglag, \, xF2qglag, \, xF1gqlag, \, xF2gqlag, \, xF3gqlag, \hfill \\ &xF1gglag, \, xF2gglag, \, xF3gglag, \, fF3gg, \, xP1polag, \, PF, \, PA, \, xPGlag, \, fPG, \, xPNFlag, \, fPNF. \hfill \\ \end{aligned}$$
  The required integrals are done numerically in the *intR* subroutine. The output of the program is put in three dimensional arrays which are called *R*(*0:nmax,2,2*).
2. At this step the evolution operator *E*^(0)^ should be calculated; this has been done in subsection “Gluon distribution at leading order”. The only difference is in the definition of the *t* parameter which should be redefined at NLO approximation. The matrices A and B which are used to get the evolution operator are like before. So in this step we can use a program similar to what has been written in subsection “Gluon distribution at leading order”. This program contains the following functions and subroutine:
  $$intp0, \, P0qq, \, P0qg, \, P0gq, \, P0gg, \, ABELO, \, NFAC, \, E0Lag$$
  After computing A and B matrices, as before we will have
  128 $$E_{n}^{(0)} (t_{NLO} ) = \sum\limits_{k = 0}^{n} {\frac{{t_{NLO}^{k} }}{{k{\mkern 1mu} !}}} \left( {A_{n}^{(k)} + B_{n}^{(k)} e^{{\lambda t_{NLO} }} } \right)$$
  In this equation, we use different values for t and we denote Eq. (128) by the function: *E0t*(*nP,IP,JP,t,A,B,nmax*). The inputs of this function are the indices of the 2 × 2 matrix elements, A and B matrices and the *t* variable. The output of the function is the numerical values of Laguerre expansion coefficients for evolution operator.
3. To get $\tilde{E}_{n}^{(1)}$ in Eq. (68) we should solve the integral in Eq. (69). For this purpose we should first solve the sum in the integrand of this equation. So Eq. (69) can be written as:
  129 $$\tilde{E}_{n}^{(1)} (t) = \int_{0}^{t} {d\tau \,e^{{ - \beta_{0} \frac{\tau }{2}}} (ERE)_{n} (t,\tau )} ,$$
  where
  130 $$(ERE)_{n} (t,\tau ) = \sum\limits_{i,j,k} {E_{i}^{(0)} (t - \tau )\,R_{j} \,E_{k}^{(0)} (\tau )} \,\delta (n - i - j - k).$$
  The $E_{n}^{(0)} (t)$ terms can be calculated using the *E0t* function [see Eq. (128)]. The *R*_*j*_ was calculated before as the *R*(*0:nmax,2,2*) array (step.1) which can be considered as the input of the program. So we should write a program which calculates the sum in Eq. (130) by taking into account the Dirac delta function and also do the matrix multiplication. The details of the program can be found in the following subroutine:
  $$SUMERE\left( {n,to,tNLO,A,B,R,SEREqq,SEREqg,SEREgq,SEREgg,nmax} \right).$$
  The outputs are the four elements of the matrix which are related to the sum in Eq. (130).
4. In this step we first calculate the integral in Eq. (129) and we would obtain the related Laguerre expansion. Then we do the loop over the order of Laguerre expansion since we are going to calculate the integral in Eq. (129) at each order *n*. The *τ* variable is obtained by generating the random number, RAN3, in the interval $[0\,,\,\,t_{NLO} ]$ so as Press et al. (1996)
  131 $$\tau = t_{NLO} \,RAN3\left( {idum} \right)$$
  Now we give the random number to the *SUMERE* subroutine. Then we call this subroutine for each generated random number in which we are able to calculate the sum in Eq. (130). To increase the precision of calculations we need to generate more random numbers which consequently increase the wall clock time. The integrals can be calculated, using the “average method” [see Eq. (73)]. We should note that four integrals are calculated which are in fact the elements of the matrix evolution operator $\tilde{E}_{n}^{(1)} (t)$. The solutions of these integrals are put in the three dimensional array *Et1*(−*2:nmax,2,2*) where the first two terms of this array for all elements of the 2 × 2 matrices are zero [see Eq. (68)].
  We continue to calculate the required expression in Eq. (68). The $E_{n}^{(1)} (t)$ term is appeared as an array by the name *E1*(*0:nmax,2,2*). This array is put inside the *n*^*th*^ loop. The term, relating to *n* = 0 is computed, using the first two terms which were introduced in Eq. (68). The other terms, relating to the Laguerre expansion at NLO approximation are obtained by iterating the loop over n, IE, JE indices.
  In the end, using this subroutine and the function *E0t* which was obtained before, we can calculate the related Laguerre expansion coefficients at the NLO approximation, based on Eq. (97). All the required mentioned tasks are gathered in a subroutine called *intE*(*En,A,B,R,ndat,nmax*). This is the most important part of the program. The outputs are the expansion coefficients of the evolution operator as 2 × 2 matrices.
5. Now by getting the Laguerre expansion for the evolution operators and the parton densities at the initial energy scale *Q*_0_^2^ = 2.56 GeV^2^, we can obtain the evolved parton densities at any desired energy scale. The process is like the one for the LO approximation. In this case we will have
  132 $$q^{( + )} (t,x) = \int_{x}^{1} {\left\{ {\sum\limits_{n = 0}^{n{\text{max}} } {E_{n,qq} (t)L_{n} \left( {\ln \frac{y}{x}} \right)} \left[ {y\tilde{q}^{( + )} (y)} \right] + \sum\limits_{n = 0}^{n{\text{max}}} {E_{n,qg} (t)L_{n} \left( {\ln \frac{y}{x}} \right)} \left[ {y\tilde{G}(y)} \right]} \right\}} \frac{dy}{{y^{2} }},$$
  133 $$G(t,x) = \int_{x}^{1} {\left\{ {\sum\limits_{n = 0}^{n{\text{max}} } {E_{n,gq} (t)L_{n} \left( {\ln \frac{y}{x}} \right)} \left[ {y\tilde{q}^{( + )} (y)} \right] + \sum\limits_{n = 0}^{n{\text{max}}} {E_{n,gg} (t)L_{n} \left( {\ln \frac{y}{x}} \right)} \left[ {y\tilde{G}(y)} \right]} \right\}} \frac{dy}{{y^{2} }}.$$
  The initial parton densities at the NLO approximations are taken from Lai et al. (1997) which are designated in Eqs. (132, 133) by tilde symbol. The list of the required functions is:
  $$qinq, \, qinU, \, qind, \, qinqUb, \, qindb, \, qinSb, \, qinG$$
  The integrals in Eqs. (132, 133) are numerically calculated, using the “average method” [see Eq. (73)]. The solution of integrals is brought into the main part of the program. The results are gluons and *q*^+^ densities (singlet sector); the singlet densities will be used in the next section to obtain the sea densities. The outputs of this program are the evolved gluon densities at different energy scales which are depicted in Fig. 10 and are compared with the CTEQ (Lai et al. 1997) and GRV (Gluck et al. 1995) parameterization groups.

If we wish to calculate the statistical error for gluon density, we should resort to Eq. (99-a). What we get for the required error at the typical energy scale *Q*^2^ = 50 GeV^2^ in the NLO approximation is  %1.78 which indicates good precision in our calculations for gluon densities (for more information, see the “Appendix”).

#### Sea quark densities at the NLO approximation

**Fig. 11:**
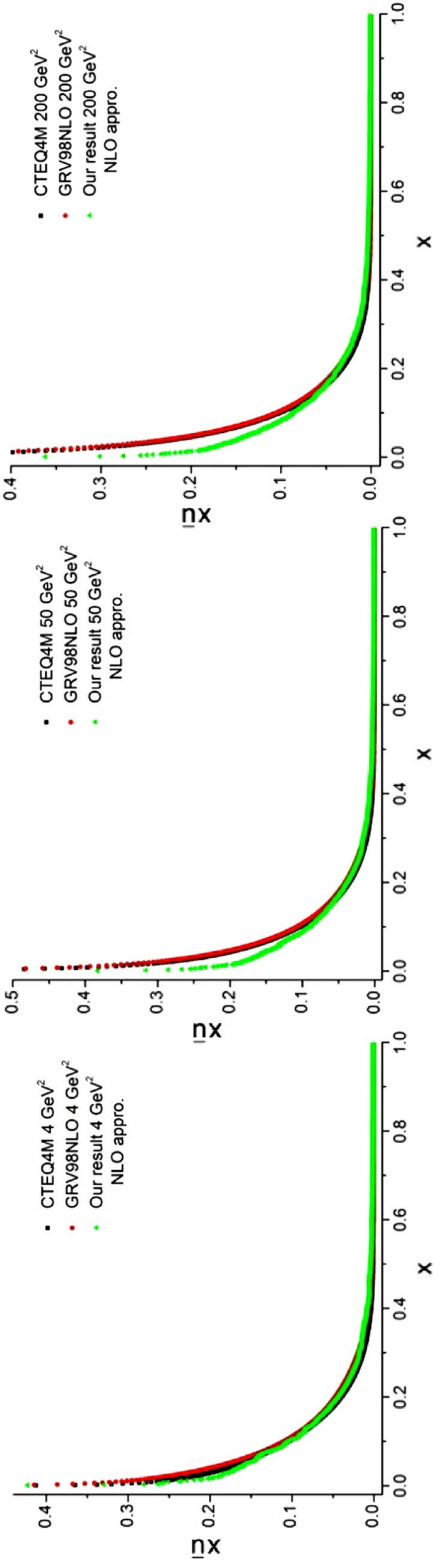
Sea u quark densities in the NLO approximation at energy scales *Q* ^2^ = 4, 50, 200 GeV^2^. Comparison with the CETQ4M and GRSV98NLO parameterization groups has also been done

**Fig. 12:**
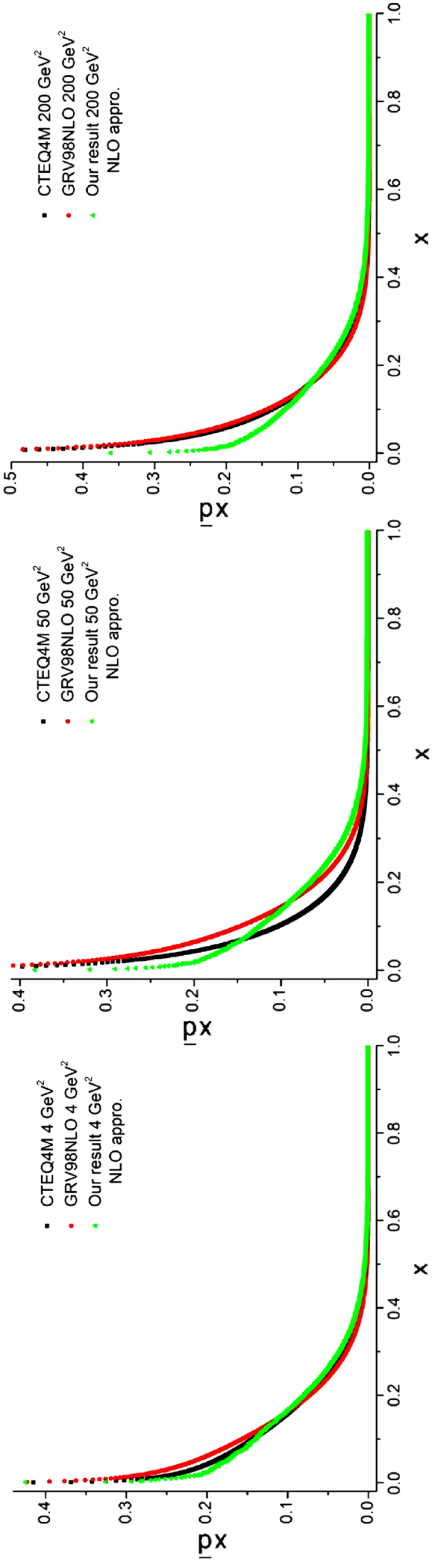
Sea d quark densities in the NLO approximation at energy scales *Q* ^2^ = 4, 50, 200 GeV^2^. Comparison with the CETQ4M and GRSV98NLO parameterization groups has also been done

**Fig. 13:**
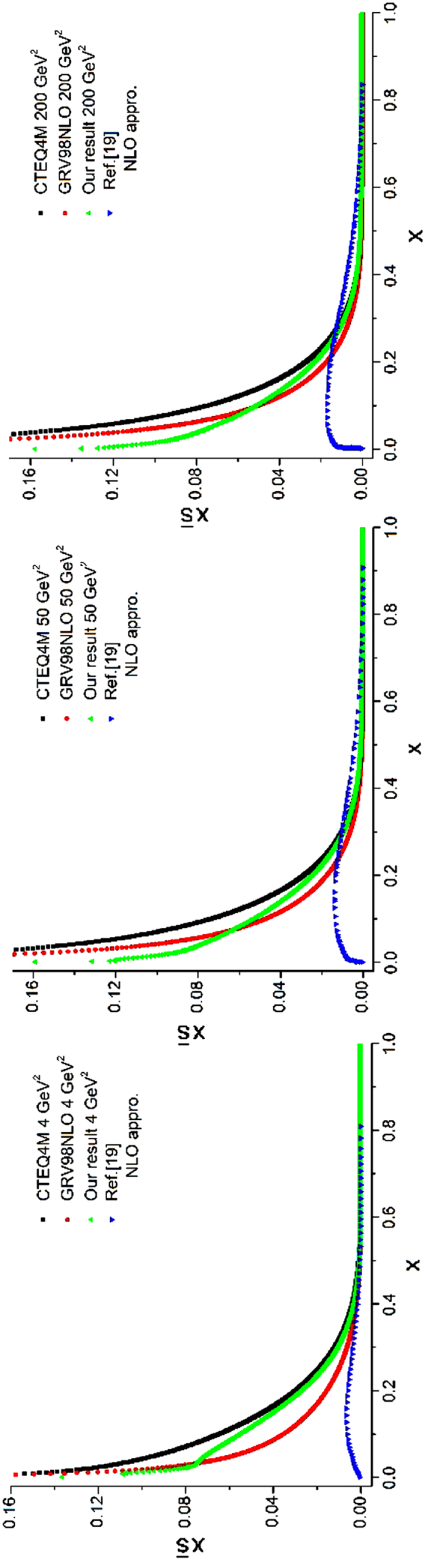
Sea s quark densities in NLO approximation at energy scales *Q* ^2^ = 4, 50, 200 GeV^2^. Comparison with CETQ4M and GRSV98NLO parameterization groups and the results from Ref. Coriano and Savkli (1999) has also been done

The objective of this subsection is to obtain the sea quark densities at the NLO approximation. The entire procedure is like what we have been done for LO approximation (see subsection “Sea quark densities at LO approximation”). The required relations to obtain the sea quark densities are given by Eqs. (123–125). Here, two programs should be run. The first program gives the gluons [see Eqs. (132, 133)]. The second program, which uses the results of the first program (*q*^(+)^) and the results of the program which gave us the *χ*_*i*_ densities [see Eq. (122) and Eq. (98)] that gave us the valence densities [$q_{i}^{( - )}$, see Eq. (98)], would yield the sea densities [see Eqs. (123–125)] at different energy scales in the NLO approximation. The results and the comparisons with the CTEQ (Lai et al. 1997) and GRV (Gluck et al. 1995) parameterization groups are presented in Figs. 11, 12 and 13 for different quark flavors.

As can be seen from Fig. 13 our results for the strange sea quark densities are in good agreement with the CETQ4 M and GRSV98NLO parameterization groups. The results from Ref. Coriano and Savkli (1999) indicate completely different behavior with respect to the fitting parameterization models as well as with respect to our results. This confirms the validity of our calculations for numerically obtaining the evolved parton densities.

To provide the statistical error for the sea parton densities we need again to resort to Eq. (99-a). The obtained errors at the typical energy scale *Q*^2^ = 50 GeV^2^ for the NLO approximation are as following (see appendix as well):

$$x\bar{U}_{{}} \to \,\,\,\% 0.17\,\,,\,\,x\bar{D}_{{}} \,\, \to \,\,\% 0.2\,\,,\,\,\,x\bar{S}\,\, \to \,\% 0.076.$$

Once again the small values for the statistical errors indicated enough precision of the employed numerical integration to evolve the parton densities

## Conclusion

In this paper, we have presented numerical solutions for the DGLAP evolution equations, based on the Laguerre polynomials expansion (Furmanski and Petroznio 1982a, b). Although people can use other methods especially in the Mellin moment space, the method which we used in this article has this specific feature that we do not need to change the space of calculations. In fact, all the computations have been done in the Bjorken x-space. We have tried to explain all the steps of performing the FORTRAN codes which produce parton densities at high energy scales. Since we have just used FORTRAN package, it means that all calculation have been done numerically. The main program can be requested from the authors via the E-mail address: a.mirjalili@yazd.ac.ir. The results are in good agreement with CETQ (Lai et al. 1997) and GRV (Gluck et al. 1995) parameterization groups. This confirms the validity of our numerical solutions for the DGLAP evolution equations. Our results for parton densities are much better than what have been represented in Coriano and Savkli (1999) especially for sea strange and gluon densities at the NLO approximation. Also the results are comparable with the results of Kobayashi et al. (1995), Schoffel (1999). A very precise technique for achieving numerical solutions for the DGLAP evolution equations can be found in Botje (2011). Also, in Kumano and Nagai (2004), a comparison between different methods, including the Laguerre polynomials expansion has been done which reveals how it is reliable to use the Laguerre polynomials to get such solutions.

 This method can be extended to evolve the polarized parton densities in a numerical way which we hope to report them in future. Further, we can evolve nucleon structure functions with two methods. One which is based on the Jacobbi polynomials expansion. The other method is related to the evolved nucleon structure function, using the evolved parton densities by Laguerre polynomials expansion as we have done in this article. Comparing these two methods provides us with the opportunity to obtain the QCD cut off parameter (Ghasempour Nesheli et al. 2015).

## Appendix

In the following tables, we listed the numerical values for the statistical error of all parton densities at different energy scales. Tables 1 and 2 are related to the LO and NLO approximations respectively.

**Table 1:** Numerical values for the statistical error of patron densities at the LO approximation

LO approximation						
*x*q	*xu*_*v*_ (%)	*xd*_*v*_ (%)	*xg* (%)	$x\bar{u}$ (%)	$x\bar{d}$ (%)	*xs* (%)
4 GeV^2^	0.699	0.313	2.05	0.186	0.197	0.077
50 GeV^2^	0.631	0.280	1.81	0.163	0.174	0.079
200 GeV^2^	0.605	0.268	1.72	0.158	0.168	0.078

**Table 2:** Numerical values for the statistical error of patron densities at the NLO approximation

NLO approximation						
*x*q	*xu*_*v*_ (%)	*xd*_*v*_ (%)	*xg* (%)	$x\bar{u}$ (%)	$x\bar{d}$ (%)	*xs * (%)
4 GeV^2^	0.760	0.329	1.78	0.168	0.196	0.076
50 GeV^2^	0.659	0.285	1.57	0.156	0.180	0.080
200 GeV^2^	0.626	0.270	1.46	0.150	0.172	0.079

## References

[CR1] Abbott LF (1979). A QCD analysis of e N deep inelastic scattering data. SLAC-PUB.

[CR2] Altarelli G, Parisi G (1977). Asymptotic freedom in parton language. Nucl Phys B.

[CR3] Arfken GB, Weber HJ (2005). Mathematical methods for physicists.

[CR4] Bjorken JD (1969). Asymptotic sum rules at infinite momentum. Phys Rev.

[CR5] Bloom ED (1969). High-energy inelastic e-p scattering at 6° and 10°. Phys Rev Lett.

[CR6] Botje M (2011). QCDNUM: fast QCD evolution and convolution. Comput Phys Commun.

[CR7] Breidenbach M (1969). Observed behavior of highly inelastic electron-proton scattering. Phys Rev Lett.

[CR8] Coriano C, Savkli C (1999). QCD evolution equations: numerical algorithms from the Laguerre expansion. Comput Phys Commun.

[CR9] Dokshitzer YL (1977). Calculation of the structure functions for deep inelastic scattering and e + e- annihilation by perturbation theory in quantum chromodynamics. Sov Phys JETP.

[CR10] Ellis RK, Stirling WJ, Webber BR (1996). QCD and collider physics, 108.

[CR11] Furmanski W, Petronzio R (1980). Singlet parton densities beyond leading order. Phys Lett B.

[CR12] Furmanski W, Petronzio R (1982). Lepton-hadron processes beyond leading order in quantum chromodynamics. Z Phys C.

[CR13] Furmanski W, Petroznio R (1982). A Method of analyzing the scaling violation of inclusive spectra in hard processes. Nucl Phys B.

[CR14] Ghasempour Nesheli A, Mirjalili A, Yazdanpanah MM (2015). Analyzing the parton densities and constructing the xF_3_ structure function using the Laguerre polynomials expansion and Monte Carlo calculations. Eur Phys J Plus.

[CR15] Gluck M, Reya E, Vogt A (1995). Dynamical parton distributions of the proton and small-*x* physics. Z Phys C.

[CR16] Gluck M, Reya E, Vogt A (1998). Dynamical parton distributions revisited. Eur Phys J C.

[CR17] Greiner W, Scharmm S, Stein E (1996). Quntum chromodynamic.

[CR18] Gribov VN, Lipatov LN (1972). Deep inelastic e p scattering in perturbation theory. Sov J Nucl Phys.

[CR19] Herrod RT, Wada S (1980). Altarelli–Parisi equation in the next-to-leading order. Phys Lett B.

[CR20] Kobayashi R, Konuma M, Kumano S (1995). FORTRAN program for a numerical solution of the nonsinglet Altarelli–Parisi equation. Comput Phys Commun.

[CR21] Kumano S, Nagai T-H (2004). Comparison of numerical solutions for Q2 evolution equations. J Comput Phys.

[CR22] Lai HL (1997). Improved parton distributions from global analysis of recent deep inelastic scattering and inclusive jet data. Phys Rev D.

[CR23] Press WH, Teukolsky SA, Vetterling WT, Flannery BP (1996). Numerical recipes in FORTRAN 90.

[CR24] Schoffel L (1999). An Elegant and fast method to solve QCD evolution equations, application to the determination of the gluon content of the pomeron. Nucl Instrum Methods.

